# *ENO* regulates tomato fruit size through the floral meristem development network

**DOI:** 10.1073/pnas.1913688117

**Published:** 2020-03-16

**Authors:** Fernando J. Yuste-Lisbona, Antonia Fernández-Lozano, Benito Pineda, Sandra Bretones, Ana Ortíz-Atienza, Begoña García-Sogo, Niels A. Müller, Trinidad Angosto, Juan Capel, Vicente Moreno, José M. Jiménez-Gómez, Rafael Lozano

**Affiliations:** ^a^Centro de Investigación en Biotecnología Agroalimentaria,Universidad de Almería, 04120 Almería, Spain;; ^b^Department of Plant Breeding and Genetics, Max Planck Institute for Plant Breeding Research, 50829 Cologne, Germany;; ^c^Instituto de Biología Molecular y Celular de Plantas, Universitat Politècnica de València–Consejo Superior de Investigaciones Científicas, 46022 Valencia, Spain;; ^d^Genome Research Group, Thünen Institute of Forest Genetics, 22927 Grosshansdorf, Germany;; ^e^Institut Jean-Pierre Bourgin, Institut National de la Recherche Agronomique (INRA), AgroParisTech, CNRS, Université Paris-Saclay, 78026 Versailles, France

**Keywords:** *Solanum lycopersicum*, fruit size, floral meristem, CLAVATA-WUSCHEL regulatory network, AP2/ERF transcription factor

## Abstract

A dramatic evolution of fruit size has accompanied the domestication and improvement of fruit-bearing crop species. In tomato (*Solanum lycopersicum*), naturally occurring cis-regulatory mutations in the genes of the CLAVATA-WUSCHEL signaling pathway have led to a significant increase in fruit size generating enlarged meristems that lead to flowers with extra organs and bigger fruits. In this work, by combining mapping-by-sequencing and CRISPR/Cas9 genome editing methods, we isolated *EXCESSIVE NUMBER OF FLORAL ORGANS* (*ENO*), an AP2/ERF transcription factor which regulates floral meristem activity. Thus, the *ENO* gene mutation gives rise to plants that yield larger multilocular fruits due to an increased size of the floral meristem. Genetic analyses indicate that *eno* exhibits synergistic effects with mutations at the *LOCULE NUMBER* (encoding *SlWUS*) and *FASCIATED* (encoding *SlCLV3*) loci, two central players in the evolution of fruit size in the domestication of cultivated tomatoes. Our findings reveal that an *eno* mutation causes a substantial expansion of *SlWUS* expression domains in a flower-specific manner. In vitro binding results show that ENO is able to interact with the GGC-box cis-regulatory element within the *SlWUS* promoter region, suggesting that ENO directly regulates *SlWUS* expression domains to maintain floral stem-cell homeostasis. Furthermore, the study of natural allelic variation of the *ENO* locus proved that a cis-regulatory mutation in the promoter of *ENO* had been targeted by positive selection during the domestication process, setting up the background for significant increases in fruit locule number and fruit size in modern tomatoes.

## Significance

Fruit-size increase is one of the major changes associated with tomato domestication, and it currently represents an important objective for breeding. Regulatory mutations at the *LOCULE NUMBER* and *FASCIATED* loci, the orthologues of the *Arabidopsis WUSCHEL* and *CLAVATA3*, have mainly contributed to enlarging fruit size by altering meristem activity. Here, we identify *ENO* as a tomato fruit regulator, which may function by regulating *WUSCHEL* gene expression to restrict stem-cell proliferation in a flower-specific manner. Our findings also show that a mutation in the *ENO* promoter was selected during domestication to establish the background for enhancing fruit size in cultivated tomatoes, denoting that transcriptional changes in key regulators have significant effects on agronomic traits.

During the domestication process, fruit-bearing crop species have largely increased their fruit size compared with those normally found in progenitor wild species. Accordingly, a large rise in fruit size has been achieved through breeding to increase the final size of floral meristems (FM) in crops such as tomato or maize (1–3). Modification of the CLAVATA (CLV)-WUSCHEL (WUS) negative feedback loop has led to this increase in meristem size. The homeodomain transcription factor WUS specifies stem-cell fate and promotes *CLV3* expression, which is a peptide ligand that binds to different plasma membrane-localized receptor complexes to initiate a signaling cascade that subsequently represses *WUS* activity (4, 5). The core signaling module of the CLV-WUS feedback loop is deeply conserved in diverse plants such as *Arabidopsis*, tomato, and maize, while dosage compensation mechanisms that operate to buffer stem-cell homeostasis in diverse lineages have diversified (6). In this way, mutations in the CLV-WUS circuit have played a relevant role in crop yield improvement of both dicots and monocots (5, 7). Thus, in tomato, mutations in the CLV3 signal peptide promote stem-cell overproliferation resulting in the development of extra organs in flowers and bigger fruits (8).

Extreme fruit size in tomato (*Solanum lycopersicum*), which evolved from the small fruited wild ancestor *S. pimpinellifolium*, is determined mainly by the number of carpels in a flower and, hence, by the final number of locules (seed compartments) forming the mature fruit (9, 10). During tomato breeding, the joint action of *fasciated* (*fas*) and *locule number* (*lc*) mutations allowed for the development of large-fruited cultivars bearing more than eight locules, in contrast with the bilocular fruits of tomato wild species and most small-fruited varieties (10, 11). The *fas* mutation is caused by a 294-kb inversion disrupting the tomato *CLV3* (*SlCLV3*) promoter (2), whereas *lc* is associated with two single-nucleotide polymorphisms (SNPs) in a putative CArG box regulatory element downstream of the tomato *WUS* (*SlWUS*) (12, 13). The *fas* and *lc* mutations are partial loss-of-function and gain-of-function alleles, respectively, and both mutations positively affect the FM size (14). A tomato mutant, *excessive number of floral organs* (*eno*), was recently reported to show alterations in FM size leading to the development of flowers with supernumerary organs and the formation of larger multilocular fruits (15). In this study, *ENO* was identified as a member of the APETALA2/Ethylene Responsive Factor (AP2/ERF) superfamily of transcription factors. Our findings suggest that ENO regulates *SlWUS* expression to restrict stem-cell proliferation in a flower-specific manner. Moreover, the analysis of genetic variation in tomato germplasm has shown that *ENO* played an important role in the increase of fruit size during tomato domestication.

## Results

### *Eno* Mutation Affects FM Size, Giving Rise to Plants with Higher Yield.

**Fig. 1.**
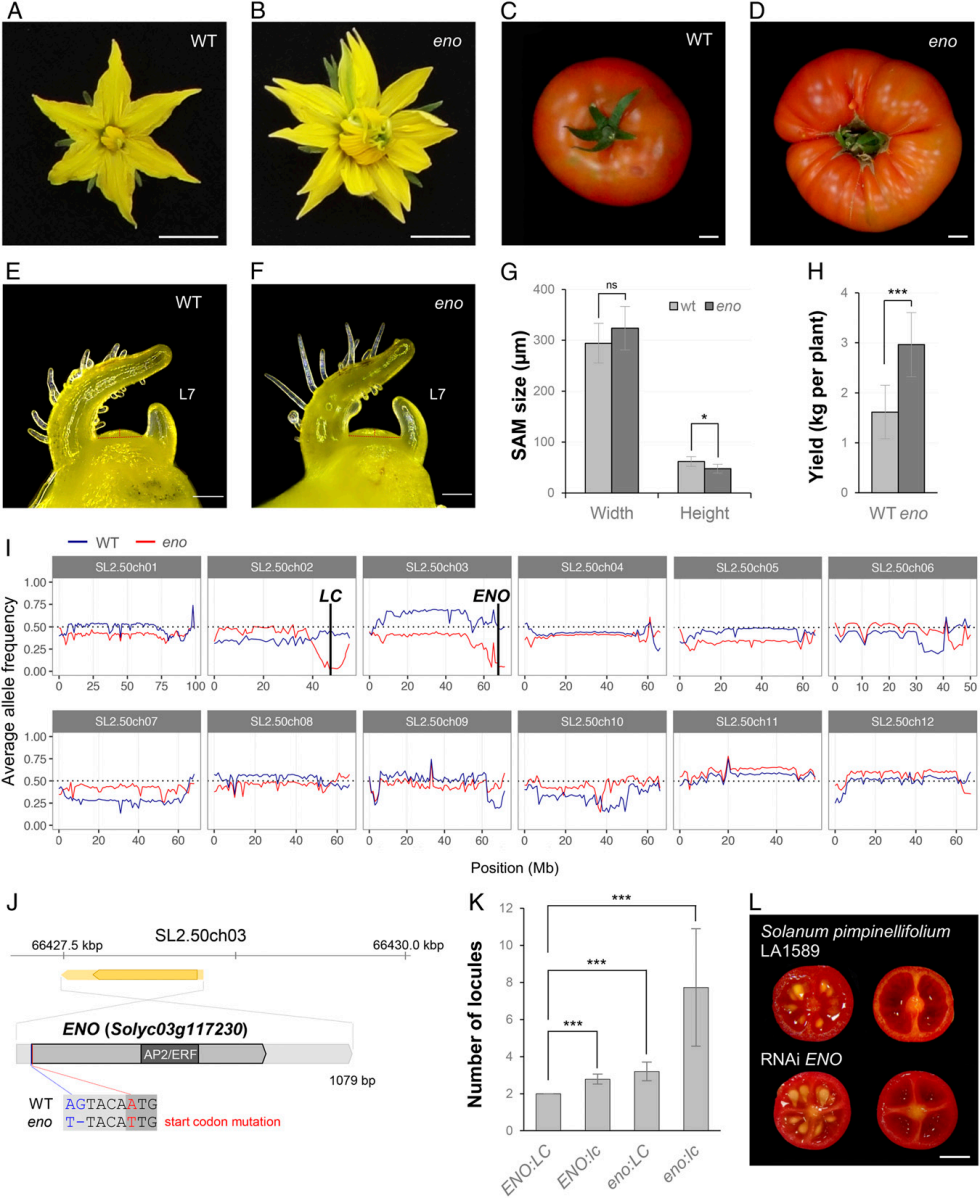
Characterization and cloning of the *eno* mutant. Representative flower (*A* and *B*) and fruit (*C* and *D*) of wild-type (WT) and *eno* mutant plants. Images of the SAM from WT (*E*) and *eno* (*F*) plants at the transition meristem stage, before forming the first floral bud (L7, leaf 7). (*G*) Quantification of SAM size from WT and *eno* plants. (*H*) Yield performance of WT and *eno* plants. (*I*) Distribution of the average allele frequency of WT (blue line) and *eno* (red line) pools grouped by chromosomes. (*J*) Positional cloning of the *ENO* gene (coding and UTRs in dark and light gray, respectively). The SNP mutation in the start codon of the *ENO* gene is marked in red, and the SNP and the InDel localized in its 5′ UTR region are shown in blue. (*K*) Number of locules for each genotyped class identified in the interspecific *eno* × LA1589 (*S. pimpinellifolium*) F2 mapping population. (*L*) RNAi-mediated knockdown of *ENO* gene in *S. pimpinellifolium* (accession LA1589). Data are means ± SD; *n* = 20 (*G*, *H*, and *K*). A two-tailed, two-sample Student’s *t* test was performed, and significant differences are represented by asterisks: ***P < 0.0001; *P < 0.01. ns, no statistically significant differences. (Scale bars, 1 cm [*A*–*D* and *L*] and 200 μm [*E* and *F*].)

Previously, we reported that *eno* mutant plants developed an increased number of floral organs and multilocular fruits (Fig. 1 *A*–*D*) (15), a phenotype reminiscent of the *CLV* gene mutants, the shoot apical meristems (SAMs) of which are enlarged (2). Based on this evidence, we examined SAM size at the transition from vegetative to reproductive growth. *eno* plants showed slightly wider and shorter SAM than the wild type (Fig. 1 *E*–*G*), in contrast to the 1.8-fold increase in the size of FM previously detected in the mutant from petal initiation and stamen primordia onward (15). Consistently with this, the increased floral organ number of *eno* is more evident in the three innermost whorls than in the outermost one (*SI Appendix*, Table S1). As a consequence of additional carpel development, *eno* plants produced larger and heavier fruits that resulted in higher yield (Fig. 1*H* and *SI Appendix*, Table S2). In addition, *eno* inflorescences were slightly more branched and contained more flowers than those developed by wild-type plants, although the number of fruits was similar in both genotypes (*SI Appendix*, Table S2). Hence, the observed phenotypes suggest a role of *ENO* in reproductive development contributing to regulation of FM size.

### *ENO* Encodes an AP2/ERF Transcription Factor.

The *eno* mutant allele arose from a transfer DNA (T-DNA) insertional mutant collection generated in the genetic background cultivar P73 (16). However, subsequent molecular analyses indicated that somaclonal variation during tissue culture rather than the T-DNA insertion was responsible for the mutant phenotype (15). To identify the mutation that underlies the *eno* locus, we performed mapping-by-sequencing on an F_2_ population derived from the cross between *eno* and a wild tomato *S. pimpinellifolium* (accession LA1589). Unlike what happened in the original tomato P73 background, where the *eno* mutant phenotype is inherited as a monogenic recessive trait (15), the 15:1 segregation ratio observed in this interspecific F_2_ population suggests that the *eno* phenotype is controlled by two independently segregating recessive genes (468 wild-type plants, 35 mutants, χ^2^ = 0.43, *P* value = 0.51). In fact, a genome-wide analysis of the allele frequencies in two pools containing 35 mutant and 50 wild-type plants revealed two genomic regions on chromosome 2 and 3 candidates to harbor the causal mutations (Fig. 1*I*). Interestingly, the region in the long arm of chromosome 2 harbors the *LC* locus (12), which is mutated in the P73 cultivar, leading to the hypothesis that *lc* and *eno* loci interact synergistically to produce extra organs and locules in flowers and fruits, respectively. Further analysis of the SNP variants on the long arm of chromosome 2 revealed that the wild-type pool was heterozygous for the *LC* locus (allele frequency 0.59), while the mutant pool was homozygous for the *lc* mutation.

**Fig. 2.**
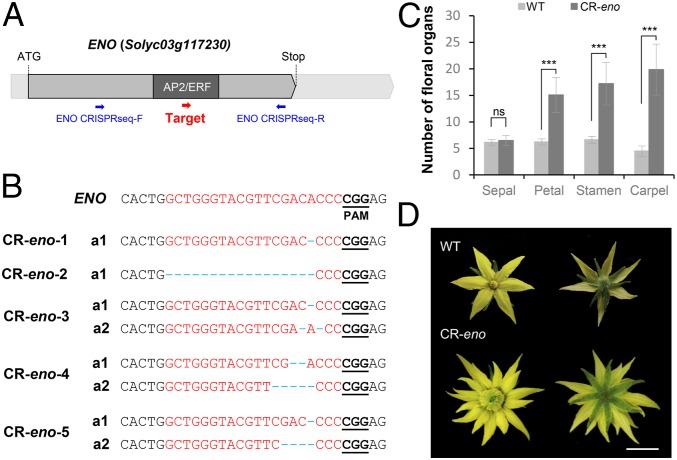
Characterization of CRISPR/Cas9-*eno* (*CR-eno*) lines. (*A*) Schematic illustrating single guide RNA targeting the *ENO* coding sequence (red arrow). Blue arrows indicate the PCR primers used to evaluate mutation type and efficiency. (*B*) *CR-eno* alleles identified by cloning and sequencing PCR products from the *ENO* targeted region from five T0 plants. Blue dashed lines indicate InDel mutations and black bold and underlined letters indicate protospacer-adjacent motif (PAM) sequences. (*C*) Quantification and statistical comparisons of floral organ number from wild-type (WT; cv. P73) and *CR-eno* flowers. Data were collected from five independent T_0_ lines. Data are means ± SDs; *n* = 10 flowers per plant. A two-tailed, two-sample Student’s *t* test was performed, and significant differences are represented by asterisks: ns, no statistically significant differences; ***P < 0.0001. (*D*) Representative flower from CRISPR/*Cas9-eno* (CR-*eno*) lines compared with wild-type (WT) one. (Scale bar, 1 cm.)

Variant analysis of a 5-Mb interval encompassing the candidate region located at the end of chromosome 3 led to the identification of a SNP in the start codon of the *Solyc03g117230* gene, as well as another SNP and one insertion/deletion (InDel) affecting its 5′ untranslated region (UTR) (Fig. 1*J*). A subsequent phylogenetic analysis showed that *Solyc03g117230* encodes a transcription factor of the AP2/ERF superfamily that belongs to the ERF subfamily group VIII (*SI Appendix*, Fig. S1). To test the identity of *Solyc03g117230* as *ENO*, we engineered knockout mutations by using the CRISPR/Cas9 system with a single guide RNA (Fig. 2*A*) in the cultivar P73 genetic background. We evaluated five independent first-generation (T_0_) diploid lines (CR-*eno*) that were homozygous or biallelic for edited mutant alleles (Fig. 2*B*). In all cases, CR-*eno* lines yield fasciated flowers and fruits resembling the phenotype observed in *eno* mutants (Fig. 2 *C* and *D* and *SI Appendix*, Table S3). Hence, our results revealed that mutations in *Solyc03g117230* (hereafter referred to as *ENO*) in combination with *lc* are responsible for the fasciation observed in flowers and fruits developed by *eno* mutant plants.

### *eno*, *lc*, and *fas* Loci Exhibit Synergistic Effects.

**Fig. 3.**
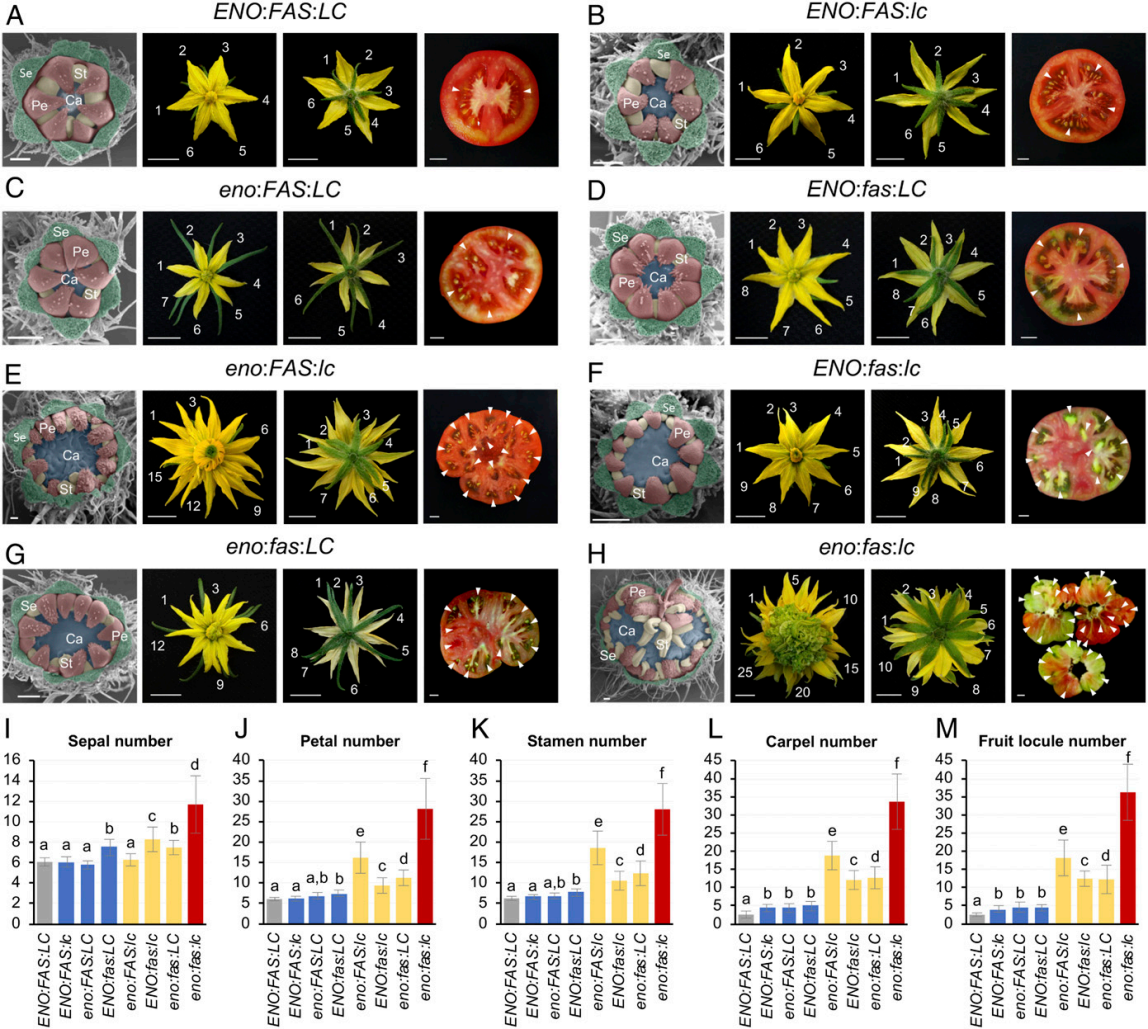
Representative floral meristems, flowers, and fruits from the different allelic combinations of *ENO*, *FAS*, and *LC loci*. (*A*) *ENO:FAS:LC*. (*B*) *ENO:FAS:lc*. (*C*) *eno:FAS:LC*. (*D*) *ENO:fas:LC*. (*E*) *eno:FAS:lc*. (*F*) *ENO:fas:lc*. (*G*) *eno:fas:LC*. (*H*) *eno:fas:lc*. Se, sepals; Pe, petals; Sta, stamens; and Ca, carpels. Note: Sepals were removed in images of floral meristems. Number of petals and sepals are specified, and arrowheads indicate locules. (Scale bars, 200 μm [floral meristems] and 1 cm [flowers and fruits].) Number of sepals (*I*), petals (*J*), stamens (*K*), carpels (*L*), and fruit locules (*M*) in wild-type plants (gray) and single (blue), double (yellow), and triple (red) mutant lines for *eno*, *fas*, and *lc* alleles. For each genotype, 10 plants were phenotyped for 10 flowers and 10 fruits (100 measurements). Values are expressed as the mean ± SD. Significant differences were calculated by pairwise comparisons of means using the least significant difference test. Values followed by the same letter (a, b, c, d, e, or f) are not statistically different (*P* < 0.05).

To determine the phenotypic effect of *eno* locus in a wild-type *LC* background, allele-specific markers for the *ENO* and *LC* loci were evaluated in the interspecific *eno* × LA1589 F_2_ mapping population. Thus, plants bearing single *lc* or *eno* mutations showed an increased number of locules with respect to wild-type ones, whereas a significant nonadditive increase in the number of locules (determined by a two-way ANOVA; *P* = 0.004) was observed in plants carrying both the *eno* and *lc* mutations (Fig. 1*K*). The effect of *eno* on locule number was additionally confirmed by an RNA interference (RNAi)-mediated knockdown of *ENO* in *S. pimpinellifolium* (LA1589), which yielded 24% of fruits with three to four locules instead of two-loculed fruits produced by wild-type plants (Fig. 1*L* and *SI Appendix*, Table S4). Likewise, in an intraspecific tomato population, *eno:LC* and *ENO:lc* genotypes gave rise to an equivalent increase in magnitude for the number of carpels and fruit locules compared with *ENO:LC* wild-type plants (Fig. 3 *A–C*, *L*, and *M*). These results support that an *eno* single locus promotes a weak increase in fruit locule number similar to that produced by an *lc* mutation.

As *lc* and *fas* loci act synergistically to increase fruit size (Fig. 3*F*) (8), we also wondered whether *eno* has genetic interaction with *fas*. To test this hypothesis, we introduced the *eno* and *fas* mutations into the wild-type *LC* background. Thus, unlike *eno* and *fas* single mutants the plants of which showed a similar feeble fasciation phenotype (Fig. 3 *C* and *D*), fasciation was synergistically enhanced in *eno*:*fas*:*LC* double-mutant plants (Fig. 3 *G* and *I–M*). Interestingly, the triple mutant for *eno*, *fas*, and *lc* dramatically increases the size of FM, giving rise to extremely fasciated flowers and fruits (Fig. 3 *H* and *I*–*M*). Although other genetic modifiers may also influence the magnitude of the observed double- and triple-mutant phenotypes, the existence of synergistic interactions indicates that *eno*, *fas*, and *lc* mutations affect different but functionally related genes, which are required to regulate FM size. As *fas* and *lc* are cis-regulatory mutations at *SlCLV3* and *SlWUS* loci, respectively (2, 12, 13), these findings suggest that *ENO* might be a component of the CLV-WUS signaling pathway; alternatively, the possibility that *ENO* acts in a parallel and convergent pathway to the CLV-WUS network not yet described in tomato cannot be ruled out.

### *ENO* Is Expressed in Shoot and Flower Meristematic Domes.

**Fig. 4.**
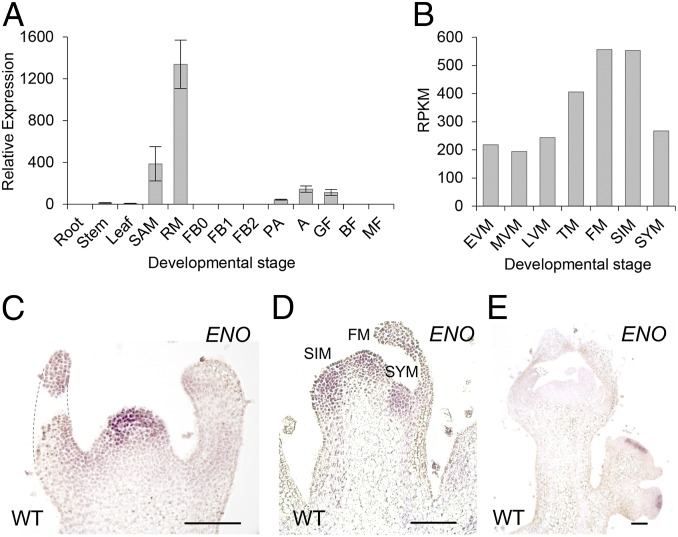
Dynamic expression of *ENO*. (*A*) qRT-PCR for *ENO* transcripts in different developmental tissues and stages. Expression was compared to that of the control *UBIQUTIN* gene. SAM, shoot apical meristem; RM, reproductive meristem; FB0, floral bud of 3.0 to 5.9 mm in length; FB1, floral bud of 6.0 to 8.9 mm in length; FB2, floral bud of 9.0 to 12 mm in length; PA, flower at preanthesis stage; *A*, flower at anthesis stage; GF, green fruit; BF, breaker fruit; MF, mature fruit. (*B*) Reads per kilobase per million reads (RPKM) values for *ENO* across vegetative and reproductive meristem stages: EVM, early vegetative meristem; MVM, middle vegetative meristem; LVM, late vegetative meristem; TM, transition meristem; FM, floral meristem; SIM, sympodial inflorescence meristem; SYM, sympodial meristem. Data were obtained from the tomato meristem maturation atlas (17). (*C*–*E*) In situ mRNA hybridization of *ENO* in vegetative and reproductive meristems of wild-type plants. (Scale bars, 100 µm.)

To further understand the function of *ENO*, we monitored its expression pattern throughout development. As expected from the phenotype of the *eno* mutation and its genetic interaction with *lc* and *fas*, we found high expression levels of *ENO* in the SAM and reproductive meristems (Fig. 4*A*). We then used the tomato meristem maturation atlas (17) to deeply assess the expression dynamic of *ENO* in meristematic tissues, which indicated that *ENO* is expressed predominantly in FM and sympodial inflorescence meristems (SIM) (Fig. 4*B*). In situ hybridization further revealed that *ENO* is expressed in the central zone of the SAM, where putative stem cells are located at the transition to the reproductive phase (Fig. 4*C*), as well as in the outermost cell layers of FM and SIM domains (Fig. 4*D*). Once flowers begin to develop, *ENO* messenger RNA (mRNA) is detected in meristematic cells within the floral buds; later, upon carpel primordia initiation, expression of *ENO* is no longer detectable (Fig. 4*E*).

### *ENO* Acts in the Genetic Network Regulating Floral Meristem Size.

**Fig. 5.**
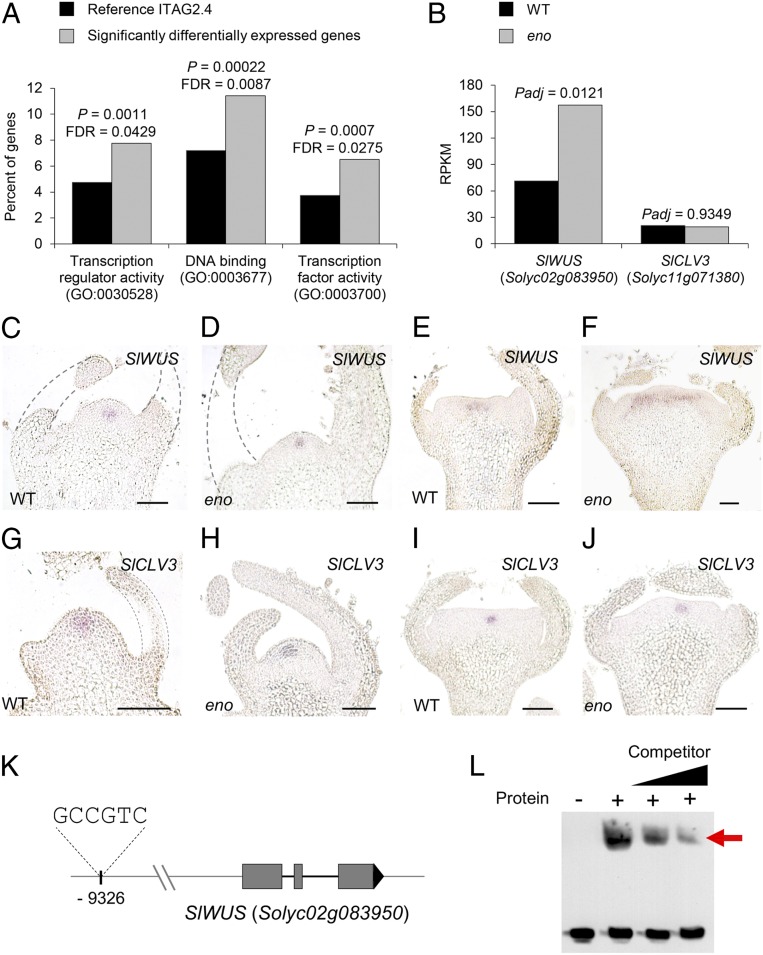
*ENO* is involved in the transcriptional regulatory network that regulates floral meristem size. (*A*) GO terms enriched among significantly differentially expressed genes between wild-type and *eno* mutant reproductive meristems using agriGO v2.0 software. A FDR < 0.05 with the Fisher statistical test and the Bonferroni multitest adjustment was used to determined enriched GO terms. (*B*) RPKM values for *SlWUS* and *SlCLV3* in wild-type (WT) and *eno* mutant. Genes with an FDR adjusted *P* value (*Padj*) < 0.05 were defined as significantly differentially expressed. (*C*–*J*) In situ mRNA hybridization of *SlWUS* (*C*–*F*) and *SlCLV3* (*G*–*J*) in shoot apical and floral meristems of wild-type and *eno* plants. (Scale bars, 100 µm.) (*K* and *L*) EMSA of ENO protein revealing binding to the SlWUS promoter. Biotinylated probe containing the theoretical ERF binding site (GCCGTC, located at −9,326 bp relative to the translational start site) on the *SlWUS* promoter (*K*) incubated with purified ENO protein (*L*). Black triangle in *L* indicates the increasing amounts (100 and 1,000) of unlabeled probe used for competition. The specific complex formed is indicated by red arrow.

We investigated the molecular signaling cascade downstream of *ENO* using RNA sequencing in reproductive meristems from *eno* and wild-type plants. This analysis identified 381 and 397 genes significantly up- and down-regulated, respectively (false discovery rate [FDR] *P* < 0.05), in the *eno* mutant relative to wild type (Dataset S1). To gain insight into the functions of these genes, we performed Gene Ontology (GO) term enrichment analysis using agriGO software (18). Particularly, a significant enrichment was found for the molecular function of transcription regulator activity (*P* = 0.0011, FDR = 0.0429), DNA binding (*P* = 0.00022, FDR = 0.0087), and transcription factor activity (*P* = 0.0007, FDR = 0.0275) (Fig. 5*A* and *SI Appendix*, Fig. S2), which suggests that *ENO* functions in a complex transcriptional network that fine-tunes the spatial and temporal regulation of genes controlling meristematic activity.

In addition, functional GO enrichment analysis using ClueGO software (19) for the corresponding *Arabidopsis* homologs of up- and down-regulated differentially expressed genes revealed 66 and 86 overrepresented GO terms, respectively (Dataset S2). Remarkably, up-regulated genes were highly enriched for GO terms associated with the meristem structural organization and meristem maintenance groups (*SI Appendix*, Fig. S3*A*). Among genes included within these groups the homologs of the *Arabidopsis WUS* (*Solyc02g083950*) and *SHOOT MERISTEMLESS* (*STM*) (*Solyc02g081120*) stand out, the latter functioning in a parallel and complementary fashion to the CLV-WUS pathway and preventing stem cells from differentiating (20). In contrast, down-regulated genes were strongly enriched for GO terms related to the specification of floral organ identity and floral organ development groups as well as, to a lesser extent, the FM determinacy and regulation of cell differentiation groups (*SI Appendix*, Fig. S3*B*). Within these groups, genes were included such as the putative homologs of the *Arabidopsis* floral homeotic genes *APETALA1* (AP1), (*Solyc05g056620*), *AP2* (*Solyc03g044300*), *AP3* (*Solyc04g081000*), *PISTILLATA* (*PI*) (*Solyc06g059970*), and *AGAMOUS* (*AG*) (*Solyc02g071730*), the latter also involved in FM determinacy (21). Taken together, these findings suggest that *ENO* loss-of-function results in prolonged FM maintenance leading to an enlargement of FM size.

The role of ENO as a transcription regulator and its genetic interaction with *lc* and *fasciated* prompted us to examine expression changes in *SlWUS* (*Solyc02g083950*) and *SlCLV3* (*Solyc11g071380*) genes in our RNA sequencing experiment. Notably, *SlWUS* expression was significantly up-regulated (fold change [FC] = 1.4) in *eno* reproductive meristems. In contrast, no significant differences were found for *SlCLV3* (Fig. 5*B* and Dataset S1). To further investigate the contribution of *ENO* to the regulation of the CLV-WUS signaling pathway, expression patterns of *SlWUS* and *SlCLV3* were examined by in situ hybridization. Thus, a similar expression pattern was observed for *SlWUS* mRNA in wild-type and *eno* SAMs (Fig. 5 *C* and *D*), while substantial expansion of *SlWUS* expression domains was found in FMs of *eno* mutants (Fig. 5 *E* and *F*). However, the *SlCLV3* mRNA domain was found to be comparable in both SAM (Fig. 5 *G* and *H*) and FM (Fig. 5 *I* and *J*) of wild-type and *eno* plants. These results suggest that *ENO* acts by regulating the spatial expression domain of *SlWUS* specifically in FM and were consistent with the *eno* mutant phenotype, which mainly shows differences in FM size. Our results also suggest that the increased FM size is produced by stem-cell overproliferation resulting from expanded *SlWUS* expression. The fact that *ENO* transcripts were detected not only in reproductive meristem but also in vegetative ones suggests that other tomato genes may have functional redundancy with *ENO* in vegetative meristems, masking the effects of its loss-of-function. In the proposed CLV-WUS signaling pathway model, WUS promotes the expression of CLV3 peptide to limit its own activity via a kinase signaling cascade mediated by plasma membrane-localized receptor complexes (5, 22). Hence, in contrast to what was observed in FM of *eno* mutants, the increase of the *SlWUS* expression domain would lead to an up-regulation of *CLV3* transcription. However, recent findings from studies of the *SlCLV3* promoter mutant allele collection have revealed a substantial complexity underlying the CLV-WUS pathway as there is not a simple linear relationship between transcriptional changes for *SlWUS* and *SlCLV3* expression levels, which is in agreement with the hypotheses that suggest a nonlinear gene dosage response for developmental regulators involved in complex transcriptional regulatory networks (8, 23).

The gene expression results indicate that ENO might specifically act in developing flowers to spatially regulate *SlWUS* expression domains. Thus, we wondered whether ENO could bind to the *SlWUS* promoter to directly regulate its transcriptional activity. The AP2 DNA binding domain of the ERF transcription factors has been shown to target GCC-related elements (GCCGGC and GCCGTC) (24). The analysis of the *SlWUS* promoter sequence revealed the existence of a GCCGTC element at position −9,326 (Fig. 5*K*). To examine whether *SlWUS* may be a direct target of ENO, the capability of ENO protein to bind to this GGC-box cis-regulatory element was tested by using an electrophoretic mobility shift assay (EMSA). A band shift was observed when the purified ENO protein was mixed with the biotin-labeled probe containing the GCCGTC motif. The presence of an excessive amount of the unlabeled probe prevented the formation of DNA-protein complexes, which indicates specific binding of ENO to this cis-regulatory element (Fig. 5*L*). Therefore, EMSA results showed that the GCCGTC motif encompassed in the *SlWUS* promoter region is a target of ENO, which indicates that ENO might function by directly regulating *SlWUS* expression domains within the complex transcriptional machinery that controls FM activity.

### Natural Allelic Variation of *ENO* Locus Affects Fruit Locule Number.

**Fig. 6.**
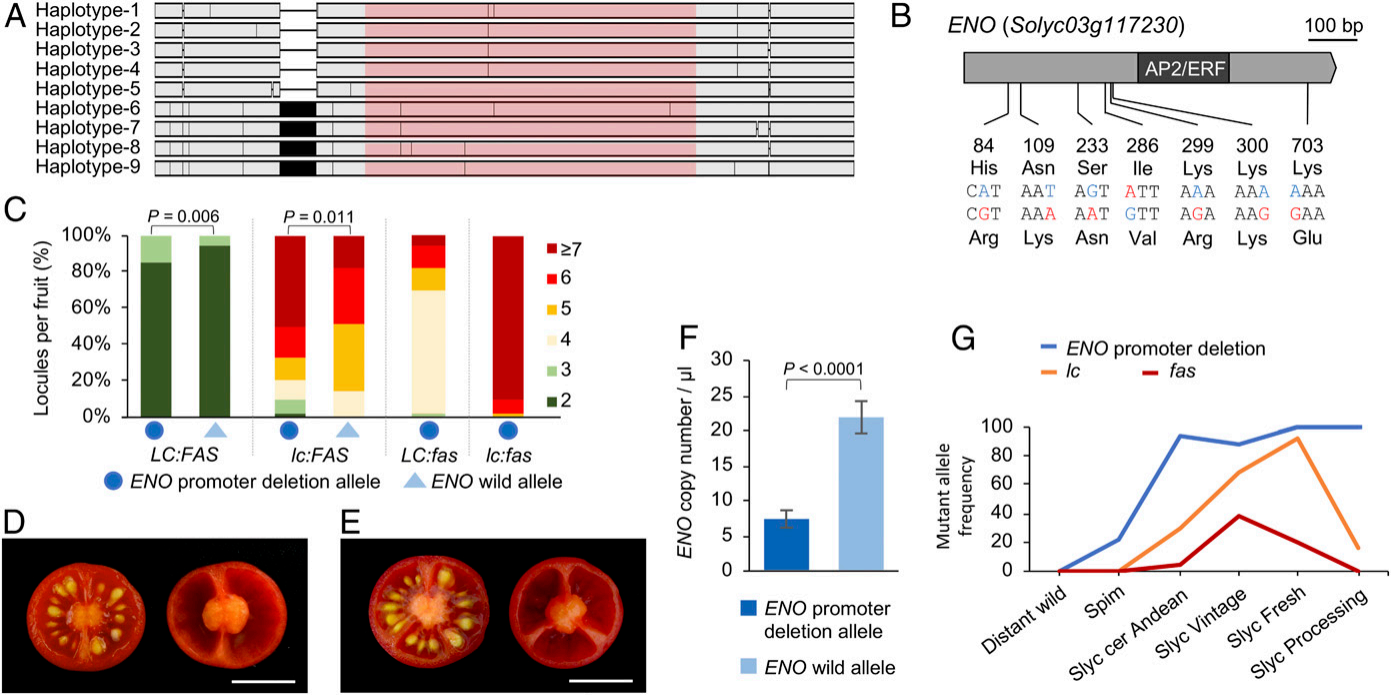
Natural allelic variation of *ENO* locus causes phenotypic variation in fruit locule number. (*A*) Multiple sequence alignment of *ENO* haplotypes identified in a set of 103 accessions producing fruits of different sizes, comprising of 92 *S. lycopersicum*, 7 *S. lycopersicum* var. cerasiforme, and 4 *S. pimpinellifolium* accessions. The *ENO* coding sequence is marked in pink. (*B*) Polymorphisms and deduced amino acid substitutions identified in the *ENO* coding sequence. (*C*) Functional effect of *ENO* promoter deletion allele on fruit locule number on the basis of genotypic information for *LC* and *FAS* loci. Fruits of *S. pimpinellifolium* accessions with the *ENO* wild allele (*D*) or the *ENO* promoter deletion allele (*E*). (Scale bars, 1 cm.) (*F*) Allele-specific *ENO* expression (copy number/μL) determined by TaqMan probe using the ddPCR assay. A two-tailed, two-sample Student’s *t* test was performed to determine significant differences between genotypes. (*G*) Frequencies of the *ENO* promoter, *lc*, and *fas* mutant alleles in phylogenetic groups representing sequential domestication steps as defined in Blanca et al. (30). Distant wild: wild tomato species; Spim: wild ancestor *S. pimpinellifolium* accessions; Slyc cer Andean: Andean accessions of *S. lycopersicum* var. cerasiforme; Slyc Vintage: *S. lycopersicum* Vintage varieties; Slyc Fresh: *S. lycopersicum* accessions for fresh market; Slyc Processing: *S. lycopersicum* accessions for industrial processing.

Previous quantitative trait locus (QTL) mapping (25–27) and genome-wide association studies (28) revealed the presence of a QTL contributing to increased fruit locule number (*lcn3.1*) at the region of the *ENO* locus. In view of the proximity of both loci, and the fact that mutations in the *ENO* gene give rise to fruits with extra locules, we hypothesized whether allelic variation at *ENO* could have contributed to the variability in fruit size present among tomato accessions. For this purpose, 1.6-kb region harboring the full-length *ENO* coding sequence was sequenced in a set of 103 accessions producing fruits of different sizes, comprising of 92 *S. lycopersicum*, 7 *S. lycopersicum* var. cerasiforme, and 4 *S. pimpinellifolium* accessions (Dataset S3). Sequence analysis identified 24 polymorphic sites and defined 9 haplotypes (*SI Appendix*, Fig. S4). Seven of these polymorphisms were detected in the *ENO* coding sequence, which resulted in one synonymous and six nonsynonymous substitutions (Fig. 6 *A* and *B*). Furthermore, we identified an 85-bp InDel annotated as a transposon-related element, which is located 107 bp upstream of the *ENO* start codon that was absent in haplotypes 1 to 5 and present in haplotypes 6 to 9 (Fig. 6*A*). To thoroughly analyze the functional effect of the detected polymorphic sites on fruit locule number, the set of accessions was additionally genotyped for *LC* and *FAS* loci (Dataset S3). Remarkably, we found a significant association between *ENO* promoter insertion polymorphism and the fruit locule number. Thus, an increase in fruit locule number was significantly associated with the absence of the 85-bp fragment (*ENO* promoter deletion allele) in both the *LC* and the *lc* background (Fig. 6*C*). It is worth highlighting that, among *S. pimpinellifolium* accessions, only fruits with two locules were found in plants with the *ENO* promoter insertion (*ENO* wild allele) (Fig. 6*D*), whereas fruits with two to three locules were found in the accession with the *ENO* promoter deletion allele (Fig. 6*E*). The functional effect of the promoter insertion polymorphism could not be evaluated in a *fas* background as tomato accessions bearing *ENO* wild allele were not found (Fig. 6*C*). From these results, we wondered about the effect of the promoter insertion polymorphism on *ENO* expression. To check this effect, allele-specific *ENO* transcript levels were measured by TaqMan probe using the Droplet Digital PCR (ddPCR) assay F1 hybrids heterozygous for the InDel mutation (haplotype-1 × haplotype-9). Notably, the copy number of the *ENO* wild allele was significantly higher (FC = 2.96) than the *ENO* promoter deletion allele (Fig. 6*F*), indicating that InDel mutation results in *ENO* expression level variation. Therefore, these results suggest that the *ENO* promoter deletion allele leads to a decreased expression of *ENO* which in turn is responsible for the increase in fruit locule number.

To further assess the evolutionary trajectory of the *ENO* promoter insertion polymorphism during tomato domestication, we analyzed this genomic region in a set of 601 resequenced accessions (29), which were clustered in phylogenetics groups representing sequential domestication steps as defined in Blanca et al. (30) (Dataset S4 and *SI Appendix*, *Materials and Methods* and Fig. S5). Results showed that the *ENO* promoter deletion allele first appeared at low frequencies in *S. pimpinellifolium* accessions, while it rose to near fixation already in the Andean *S. lycopersicum* var. cerasiforme group, the next step of domestication (Fig. 6*G*). Interestingly, all *S. lycopersicum* accessions tested contained the *ENO* promoter deletion allele, except for a few Vintage accessions that contained introgressions from wild species in the region of *ENO* (*SI Appendix*, Fig. S6). In contrast to the *ENO* promoter mutation, the *lc* and *fas* mutations arose at low frequency in the Andean *S. lycopersicum* var. cerasiforme group and varied in frequency in the course of tomato breeding depending on the group tested (Fig. 6*G*). Hence, taken together, the results show that the *ENO* promoter deletion allele arose prior to tomato domestication and increased in frequency to reach fixation in cultivated tomato, setting up the genetic environment that made significant changes in fruit size possible through selection and breeding of *lc* and *fas* mutant alleles.

## Discussion

The balance between stem-cell proliferation and differentiation is tightly regulated by a complex transcription factor network that modulates meristematic activity. This equilibrium is achieved by a negative-feedback loop involving *WUS* and *CLV* genes, which maintains meristem homeostasis. WUS is known to regulate the *CLV3* expression in a concentration-dependent manner (31). CLV3 is a secreted peptide that acts through plasma membrane-localized receptor complexes to activate a kinase signaling cascade leading to the repression of *WUS* transcription (4, 5). However, little is known about this downstream signaling pathway that finally controls *WUS* expression domains. In this context, our findings reveal that *ENO*, encoding a member of the AP2/ERF superfamily of transcription factors, is a component of the transcriptional regulatory network that specifically controls floral meristem activity, which might act to spatially limit the transcription of *SlWUS*. Overall, genetic and molecular data indicate that the *ENO* loss-of-function phenotype was due to a failure to properly repress *SlWUS* expression domains, which would most likely promote stem-cell overproliferation in FMs and finally give rise to an increase in the number of locules in tomato fruits. In agreement with these findings, the *ENO* (*Solyc03g117230*) gene has been included in a cluster of 29 genes proposed to regulate stem-cell function, which are also coexpressed with *SlWUS*. Transcripts of these genes are highly accumulated in FM whereas they diminish as the floral organ primordia are initiated (14). Within this meristematic cluster, *SlWUS* and *ENO* were the only ones showing significant genotype by developmental effects. Indeed, both genes showed a different expression pattern along FM developmental stages of *lc*, *fas*, and *lc:fas* mutants (14), which supports the functional role of ENO as a key member of the transcriptional network that regulates FM size. Likewise, the in vitro DNA-protein interaction analysis revealed that ENO is able to bind to the GCCGTC cis-regulatory element located in the *SlWUS* promoter region. Despite the fact that this DNA-protein interaction needs to be further investigated by in vivo studies, results obtained by in vitro EMSA experiments support that ENO might act directly by regulating *SlWUS* expression domains to maintain stem-cell homeostasis in a flower-specific manner.

The AP2/ERF superfamily members are classified according to the number of AP2 DNA binding domains that they contain. Thus, AP2 and ERF subfamily genes possess a double tandem-repeat and a single AP2 domain, respectively (32). Genes of the AP2 clade participate primarily in the regulation of developmental programs. For example, mutant studies indicate that the *Arabidopsis AP2* gene has many important developmental functions, including stem-cell maintenance (33) and floral development (34), whereas the other members of the AP2 group act redundantly as flowering repressors (35). However, members of the AP2 clade are likely functionally divergent outside Brassicaceae, as they control fruit development and ripening in tomato (36, 37). The ERF subfamily genes are mainly involved in the response to environmental stresses and subdivided in turn into 12 groups (32). This work’s findings revealed that *ENO* encodes a transcription factor of the ERF subfamily group VIII (*SI Appendix*, Fig. S1). Within this clade, some members involved in developmental processes have been described such as the *Arabidopsis DORNRÖSCHEN* (*DRN*) and *DORNRÖSCHEN-LIKE* (*DRNL*) genes, which affect shoot meristem development and participate in the genetic control of embryogenesis (38). Furthermore, *DRNL* expression marks floral organ founder cells, and it is hypothesized that *DRNL* contributes to positional determination for floral organ initiation (39). The *Arabidopsis* PUCHI, an AP2/ERF protein closely related to DRNL, specifies floral meristem identity and bract suppression (40), whereas the PUCHI orthologs BRANCHED SILKLESS1 (BD1) in maize (41) and FRIZZY PANICLE (FZP) in rice (42) function in floral fate determination, revealing a conserved floral function for PUCHI. Hence, the present study provides evidence of the functional role of an ERF transcription factor specifically involved in regulating floral meristematic activity.

Recent research in crop species has substantially expanded knowledge on how the regulation of meristematic activity can lead to developmental alterations with significant implications for crop improvement (5, 7). In tomato, the variation from bilocular fruit to large-fruited cultivars bearing more than eight locules has been achieved by the combinatorial effects of *lc* and *fas* loci, which synergistically increase fruit size as a result of mutations in the CLV-WUS circuit (2, 13, 43). The findings in the present work reveal that *ENO* is a regulator of tomato fruit size, which has been targeted by positive selection during the domestication process. Thus, an increase in fruit locule number was significantly associated with an 85-bp deletion in the *ENO* promoter region resulting in a reduction of its expression, which supports the important role of cis-regulatory elements in crop improvement (44). In addition, the overall evolutionary trajectory of the *ENO* promoter and *lc* and *fas* mutations during tomato domestication and breeding revealed that, while *lc* and *fas* mutations were absent in the wild tomato species, the *ENO* promoter deletion allele arose in the wild ancestor *S. pimpinellifolium* and was selected during domestication, setting up the background for significant increases in fruit size in modern tomatoes through mutations in *LC* and *FAS* loci.

Collectively, this current work highlights that much still remains to be understood about the factors controlling meristem size and that there are unsuspected regulators of meristematic activity waiting to be discovered. Our findings show the potential to increase crop productivity by tinkering with genes that help to define the expression domains of the *WUS* stem-cell identity gene. In this respect, future studies for expanding our understanding of the molecular mechanisms governing meristem size maintenance would have far-reaching implications for enhanced agricultural yields. With the availability of genome editing tools, such as CRISPR/Cas9, it is currently possible to generate new customized alleles for crop productivity optimization to meet agricultural and environmental challenges. For example, further characterization of the *SlWUS* cis-regulatory region or the identification of new components in its transcriptional regulation may provide promising targets to engineer novel weak alleles that will have beneficial effects on tomato crop improvement.

## Materials and Methods

A detailed description of plant materials, plant growth conditions, microscopy, gene expression studies, vector construction and plant transformation, bioinformatic sequence analysis, the DNA-protein interaction assay, and any associated references are available in *SI Appendix*, *Materials and Methods*.

### Data Availability.

The sequencing datasets for this study can be found in the National Center for Biotechnology Information Short Read Archive under the BioProject accession codes PRJNA503558 (45) and PRJNA495568 (46).

## Supplementary Material

Supplementary File

Supplementary File

Supplementary File

Supplementary File

Supplementary File
