# Supplementary material for: ENO regulates tomato fruit size through the floral meristem development network
Source: Proc Natl Acad Sci U S A. 2020 Mar 16;117(14):8187–95. doi: 10.1073/pnas.1913688117 (PMC7148573; doi:10.1073/pnas.1913688117)
Supplement: Supplementary File [file pnas.1913688117.sapp.pdf]

PNAS
www.pnas.org

Supplementary Information for

ENO regulates tomato fruit size through the floral meristem development network

Fernando J. Yuste-Lisbona, Antonia Fernández-Lozano, Benito Pineda, Sandra Bretones, Ana Ortíz-Atienza, Begoña García-Sogo, Niels A. Müller, Trinidad Angosto, Juan Capel, Vicente Moreno, José M. Jiménez-Gómez, Rafael Lozano

Corresponding author: Rafael Lozano
Email: rlozano@ual.es

This PDF file includes:

SI Materials and Methods
Figures S1 to S6
Tables S1 to S6
SI References

Other supplementary materials for this manuscript include the following:

Datasets S1 to S4

www.pnas.org/cgi/doi/10.1073/pnas.1913688117

SI Materials and Methods

Plant material and growth conditions. The *eno* mutant was identified from a T-DNA insertional mutant collection generated in the genetic background cultivar P73 (1); however, molecular analyses prove that somaclonal variation during tissue culture process was responsible for the mutant phenotype (2). For mapping-by-sequencing, an F_2 mapping population was generated by crossing an *eno* mutant to wild tomato *Solanum pimpinellifolium* accession LA1589 and self-fertilizing the F_1 plants. Single, double and triple mutant combinations of *lc*, *fas* and *eno* loci were generated using standard crossing schemes and were confirmed by genotyping from F_2 progeny plants. The accession 170045, homozygous for the mutant allele of *FAS* locus, was used to generate different *fas* mutant combinations. Wild and cultivated tomato accessions used for sequencing the *ENO* locus are listed in Dataset S3 and were obtained from the Institute for Mediterranean and Subtropical Horticulture "La Mayora" (IHSM-UMA-CSIC, Málaga, Spain), kindly provided by Dr. R. Fernández-Muñoz. All experiments were carried out in greenhouse conditions under natural sunlight (13 to 14h photoperiod). Daytime and night-time temperatures ranged between 26-34 °C and 20-25 °C, respectively. Standard management practices were used including regular addition of fertilizers.

Meristem size measurements. Hand-dissected shoot apical meristems (SAMs) were captured at the transition to the reproductive phase on a Leica DMS1000 digital microscope. All images were taken by using Z-series and merged to create focused images. The SAM size was measured at the maximum width between leaf primordia using Leica Application Suite v4.9 software.

Scanning-electron microscopy (SEM). Floral buds after carpel initiation stage were prepared for SEM observation as described (3). Briefly, floral buds were hand-dissected and fixed in FAEG (10% formaldehyde, 5% acetic acid, 50% absolute ethanol and 0.72% glutaraldehyde) and stored in 70% ethanol. Subsequently, tissues were dehydrated in ethanol series and dried using a critical point dryer Bal-Tec CPD 030. Finally, samples were gold coated in a Sputter Coater (Bal-Tec SCD005) and analyzed using a Hitachi S-3500N scanning electron microscope at 10 kV.

Whole genome sequencing and allele frequency analysis. Genomic DNA was extracted from young leaves by using the DNAzol® Reagent kit (Invitrogen Life Technologies, San Diego, CA, USA) and quantified using NanoDrop 2000 spectrophotometer (Thermo Scientific, Wilmington, DE, USA). Two pools were formed using equal amount of DNA from 35 mutant and 50 randomly selected wild-type plants. Libraries were generated according to the Illumina TruSeq DNA protocol and sequenced with paired-end 100 bp on the Illumina HiSeq2000 platform (Illumina, Inc., San Diego, CA, USA). The resulting short reads have been deposited in the Sequence Read Archive (SRA) database at the National Center for Biotechnology Information (NCBI) under BioProject accession number PRJNA503558. We obtained an average of 348.98 million paired-end reads per sample (*SI Appendix*, Table S5), which were aligned to the tomato genome reference sequence version 2.50 (ITAG2.4) using Bowtie2 version 2-2.0.0-b5 with default parameters (4). Duplicated reads were removed using Picard version 1.65, and InDels were realigned using GATK v2.2-8 (5). Subsequently, variants were called using GATK v2.2-8 under default settings. Filtering of variants was performed using VCFtools (6) and the following parameters: --min-alleles 2 --max-alleles 2 --min-meanDP 20 --max-meanDP 40. After filtering, the allele frequency ratio (i.e. non-reference allele counts / total allele counts) for bi-allelic variants was calculated. Finally, to identify the chromosomal region where the *eno* mutation is located, the average allele frequencies were plotted

along each chromosome using a custom script in the R environment for statistical computing (7) that uses a sliding window and step size of 1000 and 100 variants, respectively.

Phylogenetic analysis. Sequence alignment and phylogenetic reconstruction of the tomato and Arabidopsis ERF subfamily group VIII was performed with MEGA6 (8). Full-length proteins were aligned using CLUSTALW (9). The phylogenetic tree was generated by the Neighbor-Joining method, using Jones-Taylor-Thornton substitution model. A bootstrap analysis with 1000 replications was performed to confirm the reliability of the constructed phylogeny.

Generation of transgenic lines. CRISPR/Cas9 mutagenesis was performed according to the protocol describe by (10). Briefly, Breaking-Cas web software (11) was used to design the sgRNA target sequence (GCTGGGTACGTTCGACACCC) within the coding region of the *ENO* (*Solyc03g117230*) gene. The CRISPR/Cas9 construct contained the Arabidopsis U626 promoter to express the sgRNA constitutively, as well as the transcriptional unit for (human codon optimized) Cas9 plant expression driven by the 35S promoter and the kanamycin resistance gene as a selective marker. Each first-generation (T_0) transgenic plant was genotyped with primers that cover the target recognition region of the sgRNA (*SI Appendix*, Table S6). PCR products were purified for cloning into the pGEMT vector (Promega, Madison, WI, USA). A minimum of 10 clones per PCR product were sequenced. Finally, only biallelic or chimeric T_0 plants for mutant knockout alleles were phenotyped. To generate the RNA interference (RNAi) *ENO* construct, a 192 bp fragment of *Solyc03g117230* cDNA was amplified using the ENO-RNAi-F and ENO-RNAi-R primers and cloned in sense and antisense orientation into the vector pKannibal (12), which was digested with *Not*I and the resulting fragment cloned into the pART27 binary vector (13). The sequences of primers used in the generation of CRISPR/Cas9 and RNAi constructs are shown in *SI Appendix*, Table S6. Genetic transformation experiments were carried out as described (14) using *Agrobacterium tumefaciens* strain LBA4404. The ploidy levels of T_0 transgenic plants were assessed by flow cytometry following a previously described protocol (15).

PCR genotyping of *ENO*, *LC* and *FAS* loci. Genomic DNA was extracted from young leaves by using the DNAzol® Reagent kit (Invitrogen Life Technologies, San Diego, CA, USA) and quantified using NanoDrop 2000 spectrophotometer (Thermo Scientific, Wilmington, DE, USA). *ENO* locus was genotyped using a CAPS marker for PCR products generated with SNP03_60481521-F and SNP03_60481521-R primers, which amplify a 444 bp fragment of the *Solyc03g117230* gene. When digested with *Rsa*I, the 444 bp fragment was cleaved into 211 and 233 bp fragments, whereas the *eno* mutation abolishes the *Rsa*I recognition site. Genotyping of *LC* and *FAS* loci was carried out as described in Rodríguez et al. (16). The sequences of primers used are shown in *SI Appendix*, Table S6.

Quantitative Real-Time PCR. Total RNA was extracted with TRIZOL (Invitrogen Life Technologies, San Diego, CA) according to the manufacturer's instructions. Contaminating DNA was removed using the DNA-free^{TM} kit (Ambion, Austin, TX, USA). RNA quantity and quality were estimated by spectrophotometer analysis using a NanoDrop 2000 spectrophotometer (Thermo Scientific, Wilmington, DE, USA) and checked by gel electrophoresis. qRT-PCR analysis was performed as described previously (1). Briefly, first-strand cDNA was synthesized from 500 ng of total RNA using M-MuLVreverse transcriptase (Fermentas Life Sciences, Hanover, MD, USA). qRT-PCR reactions were performed using gene-specific primers (*SI Appendix*, Table S6) and the SYBR Green PCR Master Mix kit (Applied Biosystems, Foster City, CA, USA) on the 7300 Real-Time PCR System (Applied Biosystems, Foster City, CA, USA). The qRT-PCR analysis was carried out using three biological and two technical replicates. The tomato *Ubiquitine3* (*Solyc01g056940*)

gene was used as an internal control and the quantification of gene expression were performed using the ΔΔCt calculation method (17).

***In situ* hybridization.** Tissue preparation, sectioning and transcript detection for *in situ* hybridization experiments were performed as described in Lozano et al. (3). Briefly, *ENO* (*Solyc03g117230*), *SlWUS* (*Solyc11g071380*) and *SlCLV3* (*Solyc02g083950*) probes were synthesized from cDNA by using the primers listed in *SI Appendix*, Table S6 and the resulting products were cloned into the pGEM-T Easy vector (Promega). After plasmid linearization, the DIG RNA labelling mix (Roche Applied Science, Indianapolis, IN, USA) and, depending on the orientation of the insert, T7 and SP6 polymerases were used for *in vitro* transcription of antisense probes. As negative control, sense RNA probes were synthesized and hybridized to sections of tomato shoot apical and floral meristems.

RNA sequencing. For expression analyses, three biological replicates per genotype were sequenced, each one with at least 30 reproductive meristems. Total RNA from each reproductive meristem pool was isolated using TRIZOL (Invitrogen Life Technologies, San Diego, CA) following the manufacturer's instructions. Contaminating DNA was removed using the DNA-free^{TM} kit (Ambion, Austin, TX, USA). Libraries were prepared according to the Illumina TruSeq RNA protocol and sequenced with paired-end 150 bp on the Illumina HiSeq2000 platform (Illumina, Inc., San Diego, CA, USA). The resulting short reads were deposited in the SRA database at the NCBI under BioProject accession number PRJNA495568. An average of 40.26 million paired-end reads per sample was obtained (*SI Appendix*, Table S5). Reads were aligned to the tomato genome reference sequence version 2.50 (ITAG2.4) using Tophat v2.0. 6 (18) with the following parameters: --max-insertion-length 12 --max-deletion-length 12 -g 1 --library-type fr-unstranded -m 1 --read-gap-length 12 --read-edit-dist 12 --read-mismatches 12 --read-realign-edit-dist 0 --no-coverage-search --segment-mismatches 3. An average of 93% of reads were uniquely aligned to the reference genome (*SI Appendix*, Table S5). The raw number of reads per transcript were counted using the Bioconductor packages GenomicFeatures and GenomicAlignments (19). Differentially expressed genes were determined using the Wald test in the DEseq2 package (20) with the Benjamini-Hochberg multiple testing correction (21) for false discovery rate (FDR). Genes with an FDR adjusted p-value of less than 0.05 were defined as significantly up- or down- regulated. For visualization purposes, reads per kilobase per million reads (RPKM) values were calculated for each transcript and replicate. Gene Ontology (GO) term enrichment analysis of the significantly differentially expressed genes was performed using agriGO software (22) and determined by FDR < 0.05 with the Fisher statistical test and the Bonferroni multi-test adjustment. The tomato ITAG2.4 annotation release and generic GO slim ontology were used to analyze GO term enrichment. In addition, the corresponding Arabidopsis homologues of up- and down-regulated differentially expressed genes were obtained using Phytomine tool available at Phytozome (23). Then, the homologous genes were used as input to perform a GO enrichment analysis on Biological processes using the Cytoscape plug-in ClueGO (Version 2.5.5) (24). The ClueGO networks were set to 'medium' and their connectivity was based on a kappa score of 0.4. A p-value correction was carried out using the Benjamini-Hochberg method. GO terms with corrected p-value < 0.05 were considered as significant.

Electrophoretic mobility shift assay (EMSA). Full-length open reading frame of *ENO* was cloned into the pENTR/D-TOPO vector (Invitrogen Life Technologies, San Diego, CA, USA) and recombined into the pGADT7 vector (Clontech, Mountain View, CA, USA) to obtain a plasmid construct containing a phage RNA polymerase promoter (T7) for the initiation of transcription, as well as the Kozak consensus sequence to enable efficient translation. *In vitro* transcription and

translation of ENO protein were performed using the TNT® Quick Coupled Transcription/Translation System (Promega, Madison, WI, USA) according to the supplier's instructions. The binding activity of ENO to specific DNA sequence which included theoretical ERF binding site (GCCGTC) on the *SlWUS* promoter was assayed using a LightShift™ Chemiluminescent EMSA kit (Thermo Scientific, Wilmington, DE, USA). Briefly, ENO protein was incubated in a binding buffer (10 mM Tris-HCl (pH 7.5), 50 mM KCl, 1 mM dithiothreitol, 2.5% glycerol, 5mM $MgCl_2$, 50ng/µg Poly(dI-dC), 0.05% NP-40) for 20 minutes at room temperature in the presence or absence of unlabeled (double-stranded) competitor probe. The biotin-labeled dsDNA probe was then added and the incubation continued for 20 min. dsDNA biotinylated probe was generated by amplification with SlWUS_EMSA-F and SlWUS_EMSA-R biotinylated primers (*SI Appendix*, Table S6). Protein-DNA complexes were resolved on 6% native polyacrylamide gels in 0.5X TBE buffer, and the biotin-labeled probes were detected according to the instructions provided with the LightShift™ Chemiluminescent EMSA kit.

Sequencing of *ENO* in wild and cultivated tomato accessions. Genomic DNA from accessions indicated in Dataset S3 was extracted using the DNAzol® Reagent kit (Invitrogen Life Technologies, San Diego, CA, USA). A 1.6 kb genomic region harboring the full-length *ENO* coding sequence was amplified by PCR in overlapping fragments of approximately 650 bp using primers listed in *SI Appendix*, Table S6. PCR products were sequenced by Sanger technology using the BigDye® Terminator v3.1 chemistry and the Applied Biosystems™ 3500 Series Genetic Analyzer (Applied Biosystems, Foster City, CA, USA). Sequence analysis and alignments were performed using Geneious software (25).

Allele-specific PCR for *ENO* InDel mutation. Allele-specific *ENO* transcript levels were determined by TaqMan probe using Droplet Digital PCR (ddPCR) assay. cDNA of F1 hybrids heterozygous for the InDel mutation (haplotype-1 x haplotype-9) was used for ddPCR following the method described in Kamitaki et al. (26). Briefly, 250 ng/µL of cDNA sample solution was combined with 11 µL of 2X ddPCR Supermix for Probes, 1 µL of target TaqMan probe (5 µM) and 1 µl each of forward and reverse primers (18 µM) in a total volume of 20 µL. After droplet generation using a QX200™ automated droplet generator (Bio-Rad, Hercules, CA, USA), PCR was performed in a T100 Thermal Cycler (Bio-Rad, Hercules, CA, USA) using the following thermal cycling conditions: 1 cycle of 10 minutes at 95°C; 40 cycles of 30 seconds at 94°C followed by 1 minute at 60°C; 1 cycle of 10 minutes at 98°C. Samples were subsequently measured using a QX200 droplet reader (Bio-Rad, Hercules, CA, USA) with QuantaSoft Software. The ddPCR analysis was carried out using three biological and two technical replicates. Primer and probe sequences used are shown in *SI Appendix*, Table S6.

Classification of re-sequenced tomato accessions in phylogenetic groups. Whole genome short read data for 601 tomato accessions (27) were downloaded from NCBI-SRA and aligned to the *S. lycopersicum* reference genome version 2.50 (28) using Bowtie2 (4) with default parameters. Reads mapping to multiple locations were removed using SAMtools (29) (parameter -q 5), duplicated reads were removed using picard-tools MarkDuplicates (http://broadinstitute.github.io/picard) and InDels were realigned using GATK RealignerTargetCreator and IndelRealigner v3.8 (5). In order to classify the re-sequenced accessions in phylogenetic groups, we compared them with 1008 accessions classified in (30) using 8700 genome-wide SNPs genotyped with the SolCAP Infinium Chip. For this purpose, we genotyped the 601 re-sequenced accessions at the SolCAP Infinium array positions (as indicated in the ITAG2.4_solCAP.gff3 file available at ftp.solgenomics.net) using GATK UnifiedGenotyper (5) with default parameters. After merging the two datasets, only variants that were bi-allelic and whose

alleles agreed in both datasets were kept. The resulting 5856 variants were filtered to remove loci in linkage disequilibrium using the LD pruning option of PLINK with parameters --mind 0.1 --geno 0.1 --indep 50 5 2.8 (31). A phylogenetic tree was estimated from the final matrix (1536 variants in 1609 accessions) using the ape package in R and the neighbor-joining method including *S. pennellii* LA0716 as a root (32). The resulting tree was plotted using the ggtree package in R (33). Tomato accessions in the tree were classified manually having into account the previously described classification (30) and their position in the tree (Dataset S4 and *SI Appendix*, Fig. S5).

Genotyping of re-sequenced tomato accessions for calculation of the *ENO* promoter, *lc* and *fas* mutant allele frequencies. Alignments from 601 re-sequenced accessions obtained as described above were genotyped for mutations at *ENO, LC* and *FAS*. The deletion in the promoter of *ENO* was genotyped by aligning the reads from all 601 re-sequenced accessions to a tomato reference genome where the deleted nucleotides were included, and visually inspecting the region for the presence or absence of reads. For *LC*, GATK UnifiedGenotyper (5) with default parameters was used to obtain the genotype at positions SL2.50ch02:47188498 (T/C) and SL2.50ch02:47188504 (A/C). Accessions with T + A alleles at these positions were considered wild type while accessions with C + C alleles were considered mutant. The mutation at *FAS* locus was genotyped by visually inspecting the alignments at positions SL2.50ch11:54877493-54877107 and SL2.50ch11:55171147-55171482 for signals of the inversion breakpoints previously described (34). Presence of abundant reads without pair and low coverage at both breakpoints were considered as the presence of the inversion. In all cases visualization of the alignments was performed with the Integrative Genomics Viewer genome browser (35).

Twenty-one Vintage tomato accessions were found to contain the wild-allele (absence of the *ENO* promoter deletion) at *ENO* locus (Dataset S4), which could be due to a recent introgression of a wild species in the genome of cultivated tomato. To investigate this possibility, we called variants using GATK UnifiedGenotyper (5) in the region of *ENO* in alignments of 139 Vintage accessions. The frequency of non-reference (alternative) bi-allelic SNPs was calculated in windows of 1000 SNPs and steps of 500 for each accession using the R Environment for Statistical Computing (7). The analysis of these frequencies along chromosome 3 shows that all vintage accessions with the *ENO* wild-allele contain high frequencies of non-reference alleles in genomic blocks that span the region of *ENO*, in contrast to all accessions carrying the *ENO* promoter deletion allele (*SI Appendix*, Fig. S6). These large blocks of non-reference alleles represent introgressions from wild species acquired during breeding.

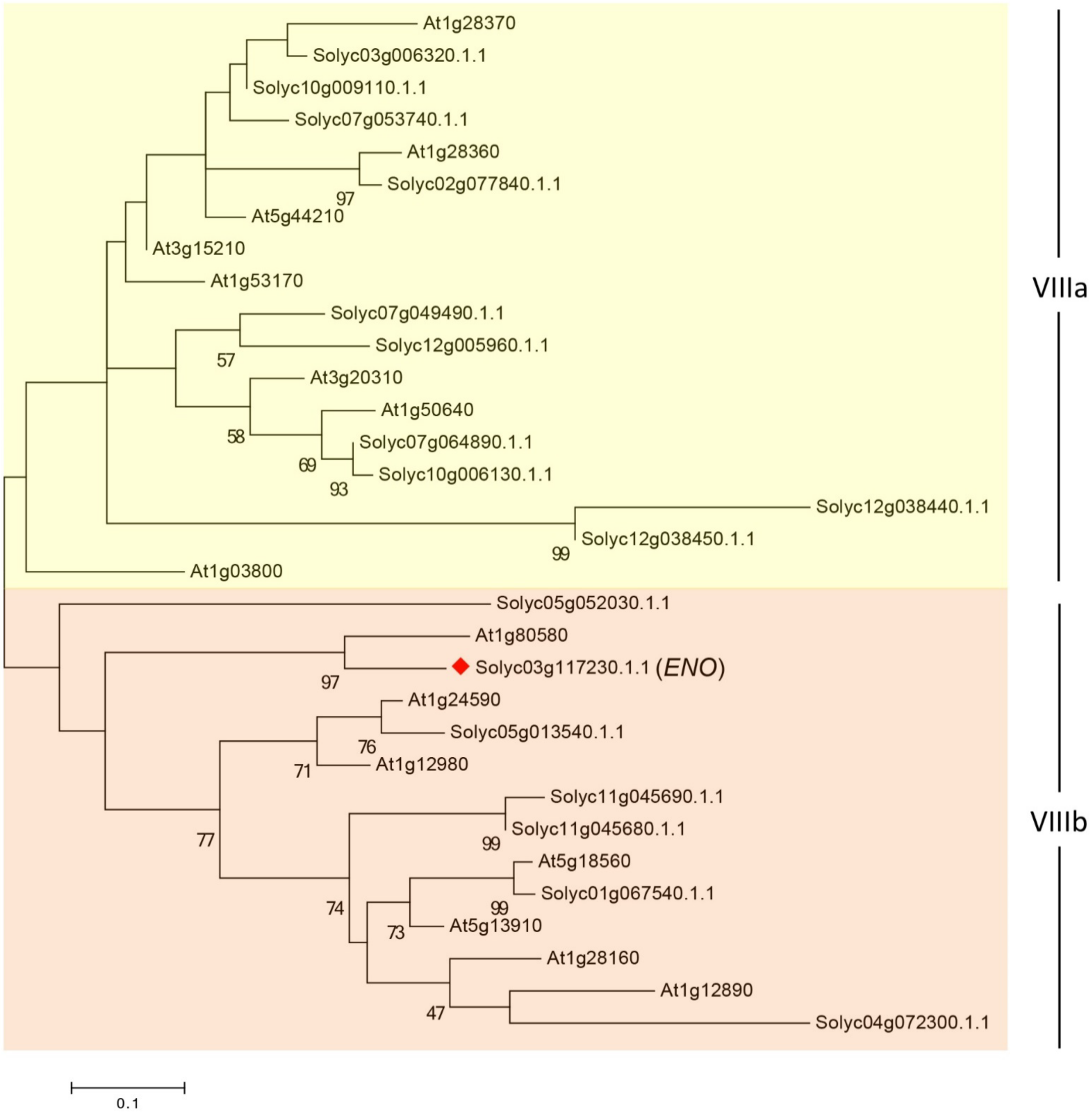

Fig. S1. Phylogenic tree of tomato and Arabidopsis ERF subfamily group VIII.

Sequence alignment and phylogenetic reconstruction of the tomato and Arabidopsis ERF subfamily group VIII was performed with MEGA6 (8). Full-length proteins were aligned using CLUSTALW (9). The phylogenetic tree was generated by the Neighbor-Joining method, using Jones-Taylor-Thornton substitution model. A bootstrap analysis with 1000 replications was performed to confirm the reliability of the constructed phylogeny. Bootstrap values of < 40% are not shown.

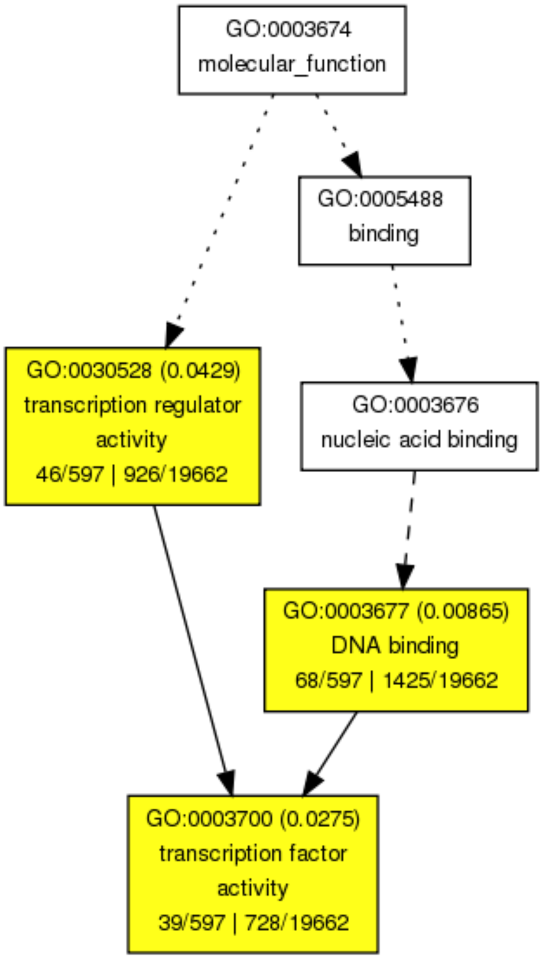

Fig. S2. Gene Ontology (GO) term enrichment analysis.

Hierarchical tree graph of enriched GO terms for significantly differentially expressed genes between wild-type and *eno* mutant reproductive meristems using agriGO v2.0 software (22). Enriched GO terms were determined by false discovery rate (FDR) < 0.05 with the Fisher statistical test and the Bonferroni multi-test adjustment.

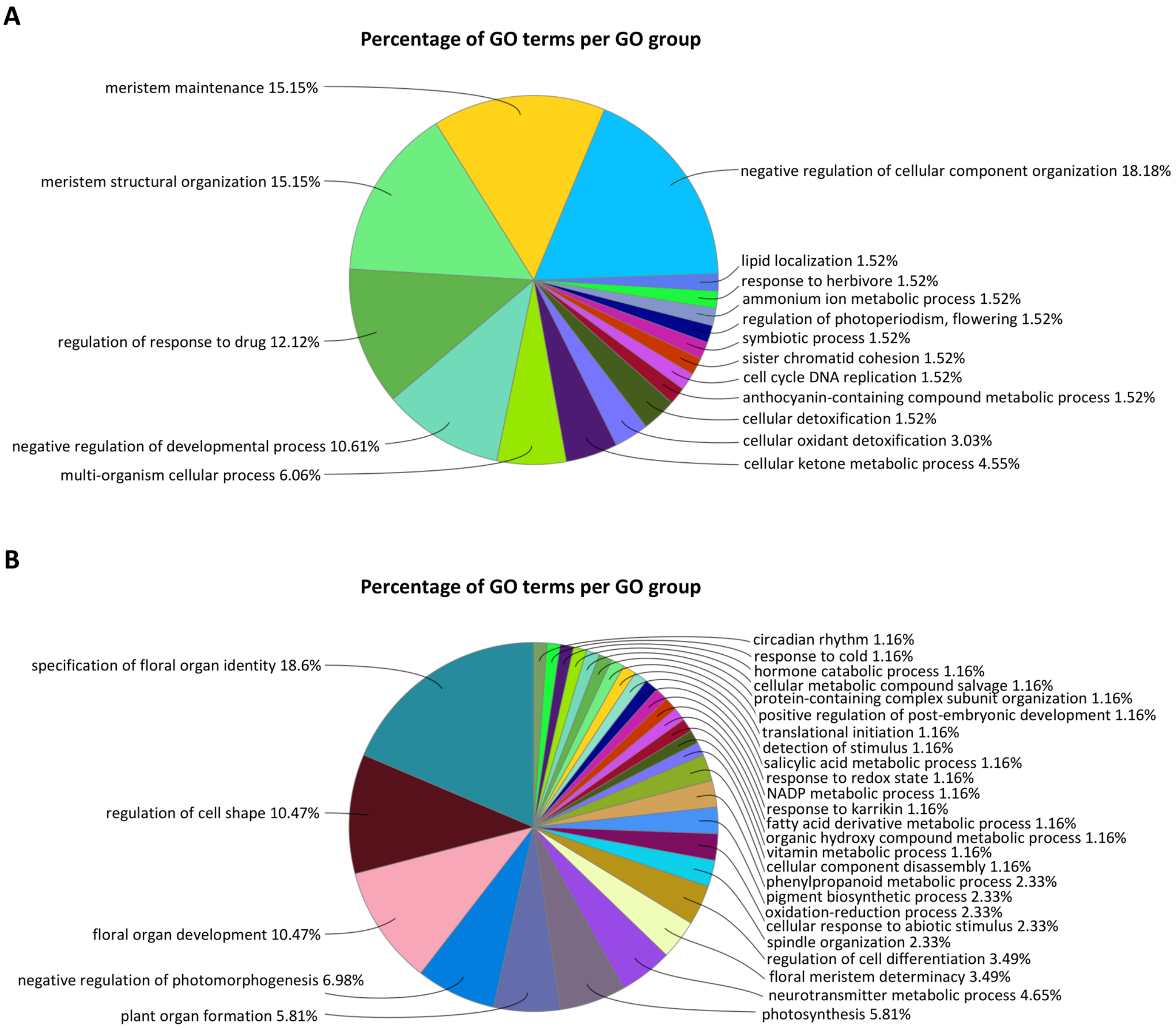

Fig. S3. Functional Gene Ontology (GO) enrichment analysis for the corresponding Arabidopsis homologues of differentially expressed genes identified in *eno* reproductive meristems.

To obtain an overview of enriched GO terms for the genes significantly (*A*) up- and (*B*) down-regulated in reproductive meristems from *eno* and wild-type plants, their putative Arabidopsis homologues were used to perform a GO enrichment analysis using the Cytoscape plug-in ClueGO (Version 2.5.5) (24). The dimension of the pie chart wedges is proportional to the number of GO terms included in each GO group, which are listed in Dataset S2.

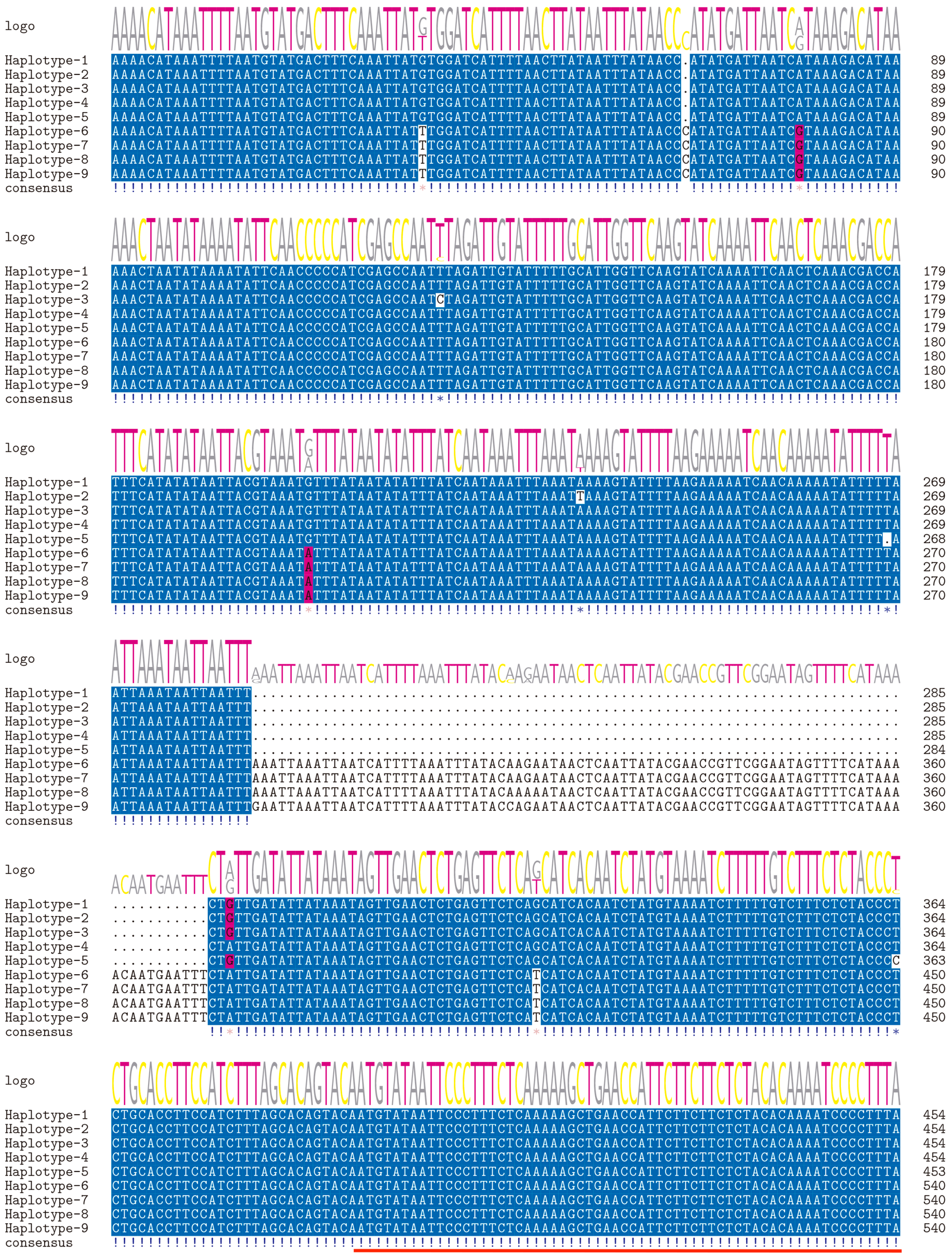

logo
Haplotype-1 AAAACATAAATTTTAATGTATGACTTTCAAATTATGTGGATCATTTTAACTTATAATTTATAACC.ATATGATTAATCATAAAGACATAA 89
Haplotype-2 AAAACATAAATTTTAATGTATGACTTTCAAATTATGTGGATCATTTTAACTTATAATTTATAACC.ATATGATTAATCATAAAGACATAA 89
Haplotype-3 AAAACATAAATTTTAATGTATGACTTTCAAATTATGTGGATCATTTTAACTTATAATTTATAACC.ATATGATTAATCATAAAGACATAA 89
Haplotype-4 AAAACATAAATTTTAATGTATGACTTTCAAATTATGTGGATCATTTTAACTTATAATTTATAACC.ATATGATTAATCATAAAGACATAA 89
Haplotype-5 AAAACATAAATTTTAATGTATGACTTTCAAATTATGTGGATCATTTTAACTTATAATTTATAACC.ATATGATTAATCATAAAGACATAA 89
Haplotype-6 AAAACATAAATTTTAATGTATGACTTTCAAATTATTTGGATCATTTTAACTTATAATTTATAACCCATATGATTAATCGTAAAGACATAA 90
Haplotype-7 AAAACATAAATTTTAATGTATGACTTTCAAATTATTTGGATCATTTTAACTTATAATTTATAACCCATATGATTAATCGTAAAGACATAA 90
Haplotype-8 AAAACATAAATTTTAATGTATGACTTTCAAATTATTTGGATCATTTTAACTTATAATTTATAACCCATATGATTAATCGTAAAGACATAA 90
Haplotype-9 AAAACATAAATTTTAATGTATGACTTTCAAATTATTTGGATCATTTTAACTTATAATTTATAACCCATATGATTAATCGTAAAGACATAA 90
consensus
logo
Haplotype-1 AAACTAATATAAAATATTCAACCCCCATCGAGCCAATTTAGATTGTATTTTTGCATTGGTTCAAGTATCAAAATTCAACTCAAACGACCA 179
Haplotype-2 AAACTAATATAAAATATTCAACCCCCATCGAGCCAATTTAGATTGTATTTTTGCATTGGTTCAAGTATCAAAATTCAACTCAAACGACCA 179
Haplotype-3 AAACTAATATAAAATATTCAACCCCCATCGAGCCAATCTAGATTGTATTTTTGCATTGGTTCAAGTATCAAAATTCAACTCAAACGACCA 179
Haplotype-4 AAACTAATATAAAATATTCAACCCCCATCGAGCCAATTTAGATTGTATTTTTGCATTGGTTCAAGTATCAAAATTCAACTCAAACGACCA 179
Haplotype-5 AAACTAATATAAAATATTCAACCCCCATCGAGCCAATTTAGATTGTATTTTTGCATTGGTTCAAGTATCAAAATTCAACTCAAACGACCA 179
Haplotype-6 AAACTAATATAAAATATTCAACCCCCATCGAGCCAATTTAGATTGTATTTTTGCATTGGTTCAAGTATCAAAATTCAACTCAAACGACCA 180
Haplotype-7 AAACTAATATAAAATATTCAACCCCCATCGAGCCAATTTAGATTGTATTTTTGCATTGGTTCAAGTATCAAAATTCAACTCAAACGACCA 180
Haplotype-8 AAACTAATATAAAATATTCAACCCCCATCGAGCCAATTTAGATTGTATTTTTGCATTGGTTCAAGTATCAAAATTCAACTCAAACGACCA 180
Haplotype-9 AAACTAATATAAAATATTCAACCCCCATCGAGCCAATTTAGATTGTATTTTTGCATTGGTTCAAGTATCAAAATTCAACTCAAACGACCA 180
consensus
logo
Haplotype-1 TTTCATATATAATTACGTAAATGTTTATAATATATTTATCAATAAATTTAAATAAAAGTATTTTAAGAAAAATCAACAAAAATATTTTTA 269
Haplotype-2 TTTCATATATAATTACGTAAATGTTTATAATATATTTATCAATAAATTTAAATTAAAGTATTTTAAGAAAAATCAACAAAAATATTTTTA 269
Haplotype-3 TTTCATATATAATTACGTAAATGTTTATAATATATTTATCAATAAATTTAAATAAAAGTATTTTAAGAAAAATCAACAAAAATATTTTTA 269
Haplotype-4 TTTCATATATAATTACGTAAATGTTTATAATATATTTATCAATAAATTTAAATAAAAGTATTTTAAGAAAAATCAACAAAAATATTTTTA 269
Haplotype-5 TTTCATATATAATTACGTAAATGTTTATAATATATTTATCAATAAATTTAAATAAAAGTATTTTAAGAAAAATCAACAAAAATATTTT.A 268
Haplotype-6 TTTCATATATAATTACGTAAATATTTATAATATATTTATCAATAAATTTAAATAAAAGTATTTTAAGAAAAATCAACAAAAATATTTTTA 270
Haplotype-7 TTTCATATATAATTACGTAAATATTTATAATATATTTATCAATAAATTTAAATAAAAGTATTTTAAGAAAAATCAACAAAAATATTTTTA 270
Haplotype-8 TTTCATATATAATTACGTAAATATTTATAATATATTTATCAATAAATTTAAATAAAAGTATTTTAAGAAAAATCAACAAAAATATTTTTA 270
Haplotype-9 TTTCATATATAATTACGTAAATATTTATAATATATTTATCAATAAATTTAAATAAAAGTATTTTAAGAAAAATCAACAAAAATATTTTTA 270
consensus
logo
Haplotype-1 ATTAAATAATTAATTT.. 285
Haplotype-2 ATTAAATAATTAATTT.. 285
Haplotype-3 ATTAAATAATTAATTT.. 285
Haplotype-4 ATTAAATAATTAATTT.. 285
Haplotype-5 ATTAAATAATTAATTT.. 284
Haplotype-6 ATTAAATAATTAATTTAAATTAAATTAATCATTTTAAATTTATACAAGAATAACTCAATTATACGAACCGTTCGGAATAGTTTTCATAAA 360
Haplotype-7 ATTAAATAATTAATTTAAATTAAATTAATCATTTTAAATTTATACAAGAATAACTCAATTATACGAACCGTTCGGAATAGTTTTCATAAA 360
Haplotype-8 ATTAAATAATTAATTTAAATTAAATTAATCATTTTAAATTTATACAAAAATAACTCAATTATACGAACCGTTCGGAATAGTTTTCATAAA 360
Haplotype-9 ATTAAATAATTAATTTGAATTAAATTAATCATTTTAAATTTATACCAGAATAACTCAATTATACGAACCGTTCGGAATAGTTTTCATAAA 360
consensus
logo
Haplotype-1 ..........CTGTTGATATTATAAATAGTTGAACTCTGAGTTCTCAGCATCACAATCTATGTAAAATCTTTTTGTCTTTCTCTACCCT 364
Haplotype-2 ..........CTGTTGATATTATAAATAGTTGAACTCTGAGTTCTCAGCATCACAATCTATGTAAAATCTTTTTGTCTTTCTCTACCCT 364
Haplotype-3 ..........CTGTTGATATTATAAATAGTTGAACTCTGAGTTCTCAGCATCACAATCTATGTAAAATCTTTTTGTCTTTCTCTACCCT 364
Haplotype-4 ..........CTATTGATATTATAAATAGTTGAACTCTGAGTTCTCAGCATCACAATCTATGTAAAATCTTTTTGTCTTTCTCTACCCT 364
Haplotype-5 ..........CTGTTGATATTATAAATAGTTGAACTCTGAGTTCTCAGCATCACAATCTATGTAAAATCTTTTTGTCTTTCTCTACCCC 363
Haplotype-6 ACAATGAATTTCTATTGATATTATAAATAGTTGAACTCTGAGTTCTCATCATCACAATCTATGTAAAATCTTTTTGTCTTTCTCTACCCT 450
Haplotype-7 ACAATGAATTTCTATTGATATTATAAATAGTTGAACTCTGAGTTCTCATCATCACAATCTATGTAAAATCTTTTTGTCTTTCTCTACCCT 450
Haplotype-8 ACAATGAATTTCTATTGATATTATAAATAGTTGAACTCTGAGTTCTCATCATCACAATCTATGTAAAATCTTTTTGTCTTTCTCTACCCT 450
Haplotype-9 ACAATGAATTTCTATTGATATTATAAATAGTTGAACTCTGAGTTCTCATCATCACAATCTATGTAAAATCTTTTTGTCTTTCTCTACCCT 450
consensus
logo
Haplotype-1 CTGCACCTTCCATCTTTAGCACAGTACAATGTATAATTCCCTTTCTCAAAAAGCTGAACCATTCTTCTTCTCTACACAAAATCCCCTTTA 454
Haplotype-2 CTGCACCTTCCATCTTTAGCACAGTACAATGTATAATTCCCTTTCTCAAAAAGCTGAACCATTCTTCTTCTCTACACAAAATCCCCTTTA 454
Haplotype-3 CTGCACCTTCCATCTTTAGCACAGTACAATGTATAATTCCCTTTCTCAAAAAGCTGAACCATTCTTCTTCTCTACACAAAATCCCCTTTA 454
Haplotype-4 CTGCACCTTCCATCTTTAGCACAGTACAATGTATAATTCCCTTTCTCAAAAAGCTGAACCATTCTTCTTCTCTACACAAAATCCCCTTTA 454
Haplotype-5 CTGCACCTTCCATCTTTAGCACAGTACAATGTATAATTCCCTTTCTCAAAAAGCTGAACCATTCTTCTTCTCTACACAAAATCCCCTTTA 453
Haplotype-6 CTGCACCTTCCATCTTTAGCACAGTACAATGTATAATTCCCTTTCTCAAAAAGCTGAACCATTCTTCTTCTCTACACAAAATCCCCTTTA 540
Haplotype-7 CTGCACCTTCCATCTTTAGCACAGTACAATGTATAATTCCCTTTCTCAAAAAGCTGAACCATTCTTCTTCTCTACACAAAATCCCCTTTA 540
Haplotype-8 CTGCACCTTCCATCTTTAGCACAGTACAATGTATAATTCCCTTTCTCAAAAAGCTGAACCATTCTTCTTCTCTACACAAAATCCCCTTTA 540
Haplotype-9 CTGCACCTTCCATCTTTAGCACAGTACAATGTATAATTCCCTTTCTCAAAAAGCTGAACCATTCTTCTTCTCTACACAAAATCCCCTTTA 540
consensus

```
logo

Haplotype-1   CCCAAATGAACCAAATCGTCACCATAATTTCTCCATTGACAACACTAATTATTCCCTCGACCCATTTTGGGATAATTACTCTGTTTCTGC   544
Haplotype-2   CCCAAATGAACCAAATCGTCACCATAATTTCTCCATTGACAACACTAATTATTCCCTCGACCCATTTTGGGATAATTACTCTGTTTCTGC   544
Haplotype-3   CCCAAATGAACCAAATCGTCACCATAATTTCTCCATTGACAACACTAATTATTCCCTCGACCCATTTTGGGATAATTACTCTGTTTCTGC   544
Haplotype-4   CCCAAATGAACCAAATCGTCACCATAATTTCTCCATTGACAACACTAATTATTCCCTCGACCCATTTTGGGATAATTACTCTGTTTCTGC   544
Haplotype-5   CCCAAATGAACCAAATCGTCACCATAATTTCTCCATTGACAACACTAATTATTCCCTCGACCCATTTTGGGATAATTACTCTGTTTCTGC   543
Haplotype-6   CCCAAATGAACCAAATCGTCACCGTAATTTCTCCATTGACAACACTAATTATTCCCTCGACCCATTTTGGGATAATTACTCTGTTTCTGC   630
Haplotype-7   CCCAAATGAACCAAATCGTCACCGTAATTTCTCCATTGACAACACTAATTATTCCCTCGACCCATTTTGGGATAATTACTCTGTTTCTGC   630
Haplotype-8   CCCAAATGAACCAAATCGTCACCGTAATTTCTCCATTGACAACACTAAATATTCCCTCGACCCATTTTGGGATAATTACTCTGTTTCTGC   630
Haplotype-9   CCCAAATGAACCAAATCGTCACCATAATTTCTCCATTGACAACACTAATTATTCCCTCGACCCATTTTGGGATAATTACTCTGTTTCTGC   630
consensus     !!!!!!!!!!!!!!!!!!!!!!!*!!!!!!!!!!!!!!!!!!!!!!!!!*!!!!!!!!!!!!!!!!!!!!!!!!!!!!!!!!!!!!!!!!

logo

Haplotype-1   TAATGAAAATTTTAATGAAAAATCCCCTGTTCTTGAAGGAATCGCTGCTGTTGTTGGAGAACATGTTCTTTTCGGTCACAGTAACAATAA   634
Haplotype-2   TAATGAAAATTTTAATGAAAAATCCCCTGTTCTTGAAGGAATCGCTGCTGTTGTTGGAGAACATGTTCTTTTCGGTCACAGTAACAATAA   634
Haplotype-3   TAATGAAAATTTTAATGAAAAATCCCCTGTTCTTGAAGGAATCGCTGCTGTTGTTGGAGAACATGTTCTTTTCGGTCACAGTAACAATAA   634
Haplotype-4   TAATGAAAATTTTAATGAAAAATCCCCTGTTCTTGAAGGAATCGCTGCTGTTGTTGGAGAACATGTTCTTTTCGGTCACAGTAACAATAA   634
Haplotype-5   TAATGAAAATTTTAATGAAAAATCCCCTGTTCTTGAAGGAATCGCTGCTGTTGTTGGAGAACATGTTCTTTTCGGTCACAGTAACAATAA   633
Haplotype-6   TAATGAAAATTTTAATGAAAAATCCCCTGTTCTTGAAGGAATCGCTGCTGTTGTTGGAGAACATGTTCTTTTCGGTCACAGTAACAATAA   720
Haplotype-7   TAATGAAAATTTTAATGAAAAATCCCCTGTTCTTGAAGGAATCGCTGCTGTTGTTGGAGAACATGTTCTTTTCGGTCACAGTAACAATAA   720
Haplotype-8   TAATGAAAATTTTAATGAAAAATCCCCTGTTCTTGAAGGAATCGCTGCTGTTGTTGGAGAACATGTTCTTTTCGGTCACAATAACAATAA   720
Haplotype-9   TAATGAAAATTTTAATGAAAAATCCCCTGTTCTTGAAGGAATCGCTGCTGTTGTTGGAGAACATGTTCTTTTCGGTCACAATAACAATAA   720
consensus     !!!!!!!!!!!!!!!!!!!!!!!!!!!!!!!!!!!!!!!!!!!!!!!!!!!!!!!!!!!!!!!!!!!!!!!!!!!!!!!!*!!!!!!!!!

logo

Haplotype-1   TAATAATAATCAAAACAAGAATGATGATCCAAATTCATCAGCAATTTCAATTCTGAAACGCACGTGTCCTGAAGAAAAGAAGAAGAATAA   724
Haplotype-2   TAATAATAATCAAAACAAGAATGATGATCCAAATTCATCAGCAATTTCAATTCTGAAACGCACGTGTCCTGAAGAAAAGAAGAAGAATAA   724
Haplotype-3   TAATAATAATCAAAACAAGAATGATGATCCAAATTCATCAGCAATTTCAATTCTGAGACGCACGTGTCCTGAAGAAAAGAAGAAGAATAA   724
Haplotype-4   TAATAATAATCAAAACAAGAATGATGATCCAAATTCATCAGCAATTTCAATTCTGAAACGCACGTGTCCTGAAGAAAAGAAGAAGAATAA   724
Haplotype-5   TAATAATAATCAAAACAAGAATGATGATCCAAATTCATCAGCAGTTTCAATTCTGAAACGCACGTGTCCTGAAGAAAAGAAGAAGAATAA   723
Haplotype-6   TAATAATAATCAAAACAAGAATGATGATCCAAATTCATCAGCAGTTTCAATTCTGAAGCGCACGTGTCCTGAAGAAAAGAAGAAGAATAA   810
Haplotype-7   TAATAATAATCAAAACAAGAATGATGATCCAAATTCATCAGCAGTTTCAATTCTGAAACGCACGTGTCCTGAAGAAAAGAAGAAGAATAA   810
Haplotype-8   TAATAATAATCAAAACAAGAATGATGATCCAAATTCATCAGCAGTTTCAATTCTGAAACGCACGTGTCCTGAAGAAAAGAAGAAGAATAA   810
Haplotype-9   TAATAATAATCAAAACAAGAATGATGATCCAAATTCATCAGCAGTTTCAATTCTGAAACGCACGTGTCCTGAAGAAAAGAAGAAGAATAA   810
consensus     !!!!!!!!!!!!!!!!!!!!!!!!!!!!!!!!!!!!!!!!!!!*!!!!!!!!!!!!**!!!!!!!!!!!!!!!!!!!!!!!!!!!!!!!!

logo

Haplotype-1   CAATAATGTTCAATCTGTGGAGAAGAGTTACAGAGGAGTGAGAAAGAGGCCATGGGGGAGATGGTCAGCGGAGATTCGCGACCGTATAGG   814
Haplotype-2   CAATAATGTTCAATCTGTGGAGAAGAGTTACAGAGGAGTGAGAAAGAGGCCATGGGGGAGATGGTCAGCGGAGATTCGCGACCGTATAGG   814
Haplotype-3   CAATAATGTTCAATCTGTGGAGAAGAGTTACAGAGGAGTGAGAAAGAGGCCATGGGGGAGATGGTCAGCGGAGATTCGCGACCGTATAGG   814
Haplotype-4   CAATAATGTTCAATCTGTGGAGAAGAGTTACAGAGGAGTGAGAAAGAGGCCATGGGGGAGATGGTCAGCGGAGATTCGCGACCGTATAGG   814
Haplotype-5   CAATAATGTTCAATCTGTGGAGAAGAGTTACAGAGGAGTGAGAAAGAGGCCATGGGGGAGATGGTCAGCGGAGATTCGCGACCGTATAGG   813
Haplotype-6   CAATAATGTTCAATCTGTGGAGAAGAGTTACAGAGGAGTGAGAAAGAGGCCATGGGGGAGATGGTCAGCGGAGATTCGCGACCGTATAGG   900
Haplotype-7   CAATAATGTTCAATCTGTGGAGAAGAGTTACAGAGGAGTGAGAAAGAGGCCATGGGGGAGATGGTCAGCGGAGATTCGCGACCGTATAGG   900
Haplotype-8   CAATAATGTTCAATCTGTGGAGAAGAGTTACAGAGGAGTGAGAAAGAGGCCATGGGGGAGATGGTCAGCGGAGATTCGCGACCGTATAGG   900
Haplotype-9   CAATAATGTTCAATCTGTGGAGAAGAGTTACAGAGGAGTGAGAAAGAGGCCATGGGGGAGATGGTCAGCGGAGATTCGCGACCGTATAGG   900
consensus     !!!!!!!!!!!!!!!!!!!!!!!!!!!!!!!!!!!!!!!!!!!!!!!!!!!!!!!!!!!!!!!!!!!!!!!!!!!!!!!!!!!!!!!!!!

logo

Haplotype-1   GCGGTGCAGACACTGGCTGGGTACGTTCGACACCCCGGAGGAGGCGGCGCGTGCGTACGACGCGGCGGCGAGGTGGCTGAGAGGTTCAAA   904
Haplotype-2   GCGGTGCAGACACTGGCTGGGTACGTTCGACACCCCGGAGGAGGCGGCGCGTGCGTACGACGCGGCGGCGAGGTGGCTGAGAGGTTCAAA   904
Haplotype-3   GCGGTGCAGACACTGGCTGGGTACGTTCGACACCCCGGAGGAGGCGGCGCGTGCGTACGACGCGGCGGCGAGGTGGCTGAGAGGTTCAAA   904
Haplotype-4   GCGGTGCAGACACTGGCTGGGTACGTTCGACACCCCGGAGGAGGCGGCGCGTGCGTACGACGCGGCGGCGAGGTGGCTGAGAGGTTCAAA   904
Haplotype-5   GCGGTGCAGACACTGGCTGGGTACGTTCGACACCCCGGAGGAGGCGGCGCGTGCGTACGACGCGGCGGCGAGGTGGCTGAGAGGTTCAAA   903
Haplotype-6   GCGGTGCAGACACTGGCTGGGTACGTTCGACACCCCGGAGGAGGCGGCGCGTGCGTACGACGCGGCGGCGAGGTGGCTGAGAGGTTCAAA   990
Haplotype-7   GCGGTGCAGACACTGGCTGGGTACGTTCGACACCCCGGAGGAGGCGGCGCGTGCGTACGACGCGGCGGCGAGGTGGCTGAGAGGTTCAAA   990
Haplotype-8   GCGGTGCAGACACTGGCTGGGTACGTTCGACACCCCGGAGGAGGCGGCGCGTGCGTACGACGCGGCGGCGAGGTGGCTGAGAGGTTCAAA   990
Haplotype-9   GCGGTGCAGACACTGGCTGGGTACGTTCGACACCCCGGAGGAGGCGGCGCGTGCGTACGACGCGGCGGCGAGGTGGCTGAGAGGTTCAAA   990
consensus     !!!!!!!!!!!!!!!!!!!!!!!!!!!!!!!!!!!!!!!!!!!!!!!!!!!!!!!!!!!!!!!!!!!!!!!!!!!!!!!!!!!!!!!!!!

logo

Haplotype-1   AGCTCGAACGAATTTCCAGATTCCTCCGATTGTCCCTCTGCCTACTTCACCAACATCCACGTCATCATCTTCAAATTCATCTCGCGAAAT   994
Haplotype-2   AGCTCGAACGAATTTCCAGATTCCTCCGATTGTCCCTCTGCCTACTTCACCAACATCCACGTCATCATCTTCAAATTCATCTCGCGAAAT   994
Haplotype-3   AGCTCGAACGAATTTCCAGATTCCTCCGATTGTCCCTCTGCCTACTTCACCAACATCCACGTCATCATCTTCAAATTCATCTCGCGAAAT   994
Haplotype-4   AGCTCGAACGAATTTCCAGATTCCTCCGATTGTCCCTCTGCCTACTTCACCAACATCCACGTCATCATCTTCAAATTCATCTCGCGAAAT   994
Haplotype-5   AGCTCGAACGAATTTCCAGATTCCTCCGATTGTCCCTCTGCCTACTTCACCAACATCCACGTCATCATCTTCAAATTCATCTCGCGAAAT   993
Haplotype-6   AGCTCGAACGAATTTCCAGATTCCTCCGATTGTCCCTCTGCCTACTTCACCAACATCCACGTCATCATCTTCAAATTCATCTCGCGAAAT   1080
Haplotype-7   AGCTCGAACGAATTTCCAGATTCCTCCGATTGTCCCTCTGCCTACTTCACCAACATCCACGTCATCATCTTCAAATTCATCTCGCGAAAT   1080
Haplotype-8   AGCTCGAACGAATTTCCAGATTCCTCCGATTGTCCCTCTGCCTACTTCACCAACATCCACGTCATCATCTTCAAATTCATCTCGCGAAAT   1080
Haplotype-9   AGCTCGAACGAATTTCCAGATTCCTCCGATTGTCCCTCTGCCTACTTCACCAACATCCACGTCATCATCTTCAAATTCATCTCGCGAAAT   1080
consensus     !!!!!!!!!!!!!!!!!!!!!!!!!!!!!!!!!!!!!!!!!!!!!!!!!!!!!!!!!!!!!!!!!!!!!!!!!!!!!!!!!!!!!!!!!!
```

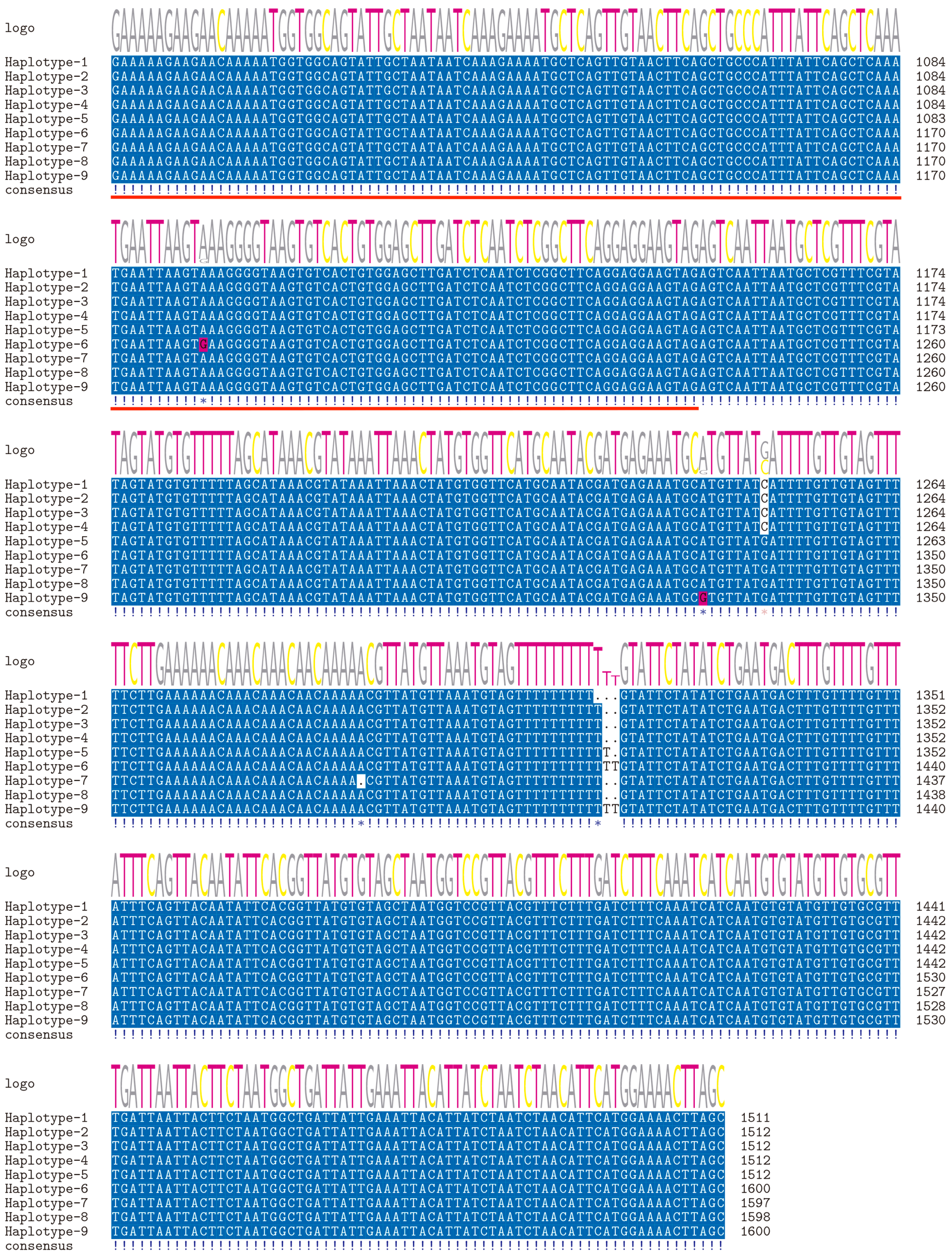

Fig. S4. Multiple sequence alignment of the *ENO* haplotypes.

The 1.6 kb of DNA sequences from the nine *ENO* haplotypes were aligned by using CLUSTALW (9) implemented in the msa Bioconductor R package (36). The *ENO* coding sequence is indicated with a red line. Exclamation marks (!) indicate fully conserved residues, while asterisks (*) indicate polymorphic sites.

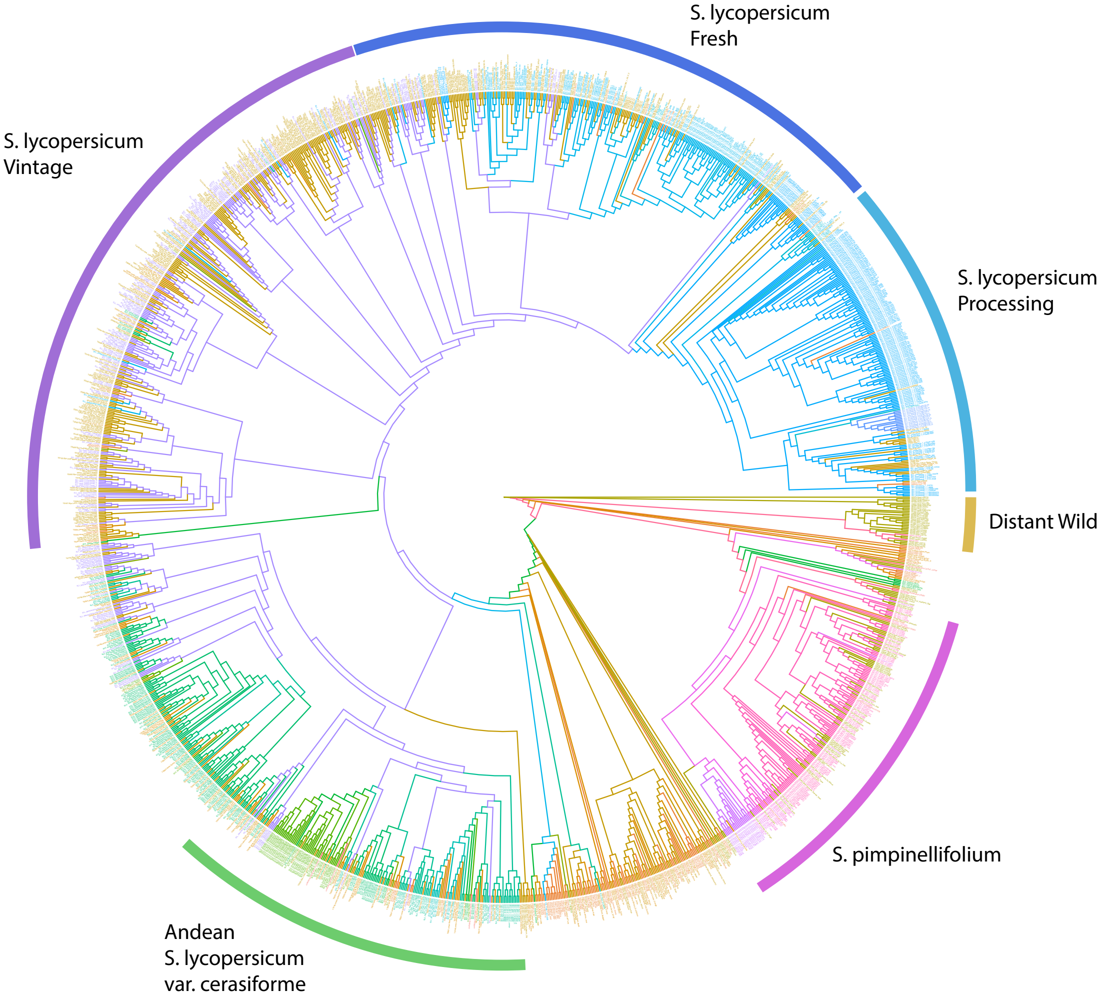

Fig. S5. Phylogenetic tree of 1609 tomato accessions.

The tree was constructed with the neighbor-joining method using 1536 genome-wide SNPs. Data was obtained from (27, 30). Colors in the branches and accessions represent the phylogenetic groups defined in by (30). All re-sequenced accessions are colored in gold. Details on groups and accessions can be found in Dataset S4. Branches of the tree used in the evolutionary analysis of *ENO* are marked by circular lines outside the tree.

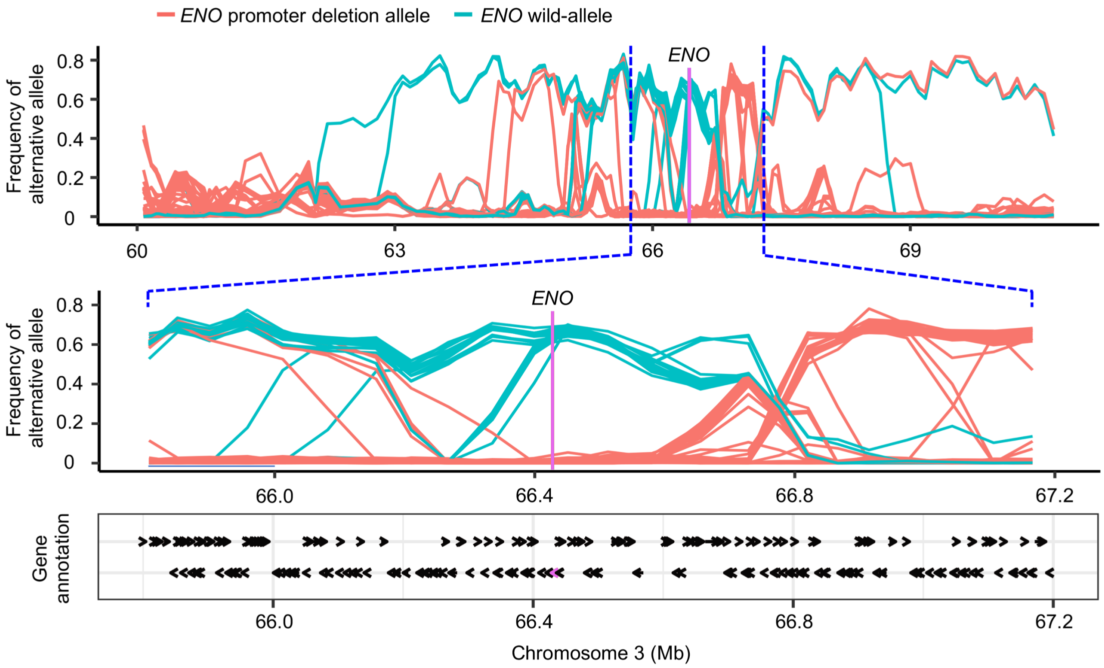

Fig. S6. Frequency of non-reference SNPs in vintage tomatoes in the region of *ENO*.

The frequency of non-reference (alternative) bi-allelic SNPs was calculated in 139 vintage tomato accessions in windows of 1000 SNPs and steps of 500. Accessions with the deletion in the promoter of *ENO* are colored in red, while accessions without the deletion colored in blue. Blocks of high frequencies of non-reference alleles are indicative of introgressions from wild species that have occurred during breeding.

Table S1. Quantification of floral organ number in wild-type (cv. P73) and *eno* plants.

Genotype	Floral organ number			
	Sepal	Petal	Stamen	Carpel
cv. P73	6.1 ± 0.5	6.2 ± 0.6	6.6 ± 0.6	4.5 ± 1.0
eno	6.3 ± 0.6 ns	16.2 ± 3.8***	18.6 ± 4.1***	18.9 ± 3.9***

For each genotype, 150 measurements (5 samples × 30 plants) were taken. Values are expressed as the mean ± standard deviation. Significant differences from wild-type were calculated by two-tailed Student's t-test and are denoted by asterisks: ****P* < 0.0001; ***P* < 0.001; **P* < 0.01. ns, no statistically significant differences.

Table S2. Quantification of inflorescence branching, flower and fruit numbers, fruit locule number and yield in wild-type (cv. P73) and *eno* plants.

Genotype	Branching	Flower number	Fruit number	Fruit locule number	Yield (kg)
cv. P73	0.42 ± 0.2	29.3 ± 4.7	19.7 ± 4.5	4.2 ± 0.9	1.6 ± 0.5
eno	2.03 ± 2.3***	40.2 ± 7.0*	18.6 ± 3.2 ns	18.2 ± 4.9***	3.0 ± 0.6***

The first four inflorescences of 30 plants were evaluated, quantifying the number of branching per inflorescence, the total number of flowers and fruits, as well as yield. For each genotype, 150 measurements were taken for determining the fruit locule number (5 samples × 30 plants). Significant differences from wild-type were calculated by two-tailed Student's t-test and are denoted by asterisks: ***P < 0.0001; **P < 0.001; *P < 0.01. ns, no statistically significant differences.

Table S3. Phenotypic characterization of CRISPR/Cas9-*eno* (CR-*eno*) lines.

Genotype	Floral organ number				Fruit locule number
	Sepal	Petal	Stamen	Carpel	
cv. P73	6.1 ± 0.5	6.2 ± 0.6	6.6 ± 0.6	4.5 ± 1.0	4.2 ± 0.9
CR-*eno*-1	6.4 ± 0.5 ns	13.0 ± 1.8***	15.5 ± 3.8***	16.6 ± 3.7***	16.6 ± 4.9***
CR-*eno*-2	6.5 ± 0.8 ns	18.1 ± 2.6***	18.7 ± 3.0***	23.0 ± 4.1***	23.1 ± 5.3***
CR-*eno*-3	6.4 ± 0.5 ns	15.5 ± 3.1***	17.5 ± 3.4***	21.4 ± 4.0***	23.5 ± 3.5***
CR-*eno*-4	5.8 ± 0.6 ns	15.3 ± 1.2***	16.8 ± 2.0***	18.8 ± 2.4***	19.4 ± 4.8***
CR-*eno*-5	6.6 ± 0.8 ns	13.1 ± 2.3***	13.8 ± 2.3***	16.8 ± 4.0***	17.7 ± 3.1***

Five independent T_0 diploid lines (in cv. P73 background) homozygous or heterozygous for mutant alleles were phenotyped for 10 flowers and 10 fruits each. Values are expressed as the mean ± standard deviation. Significant differences from wild-type were calculated by two-tailed Student's t-test and are represented by asterisks: ***P < 0.0001; **P < 0.001; *P < 0.01. ns, no statistically significant differences.

Table S4. Phenotypic characterization of RNAi *ENO* lines.

Genotype	Floral organ number				Fruit locule number
	Sepal	Petal	Stamen	Carpel	
LA1587	5.0 ± 0.0	5.0 ± 0.0	5.0 ± 0.0	2.0 ± 0.0	2.0 ± 0.0
Sp RNAi *ENO*-1	5.0 ± 0.0 ns	5.0 ± 0.0 ns	5.0 ± 0.0 ns	2.3 ± 0.4*	2.3 ± 0.5*
Sp RNAi *ENO*-2	5.0 ± 0.0 ns	5.1 ± 0.3 ns	5.0 ± 0.0 ns	2.3 ± 0.5*	2.3 ± 0.4*
Sp RNAi *ENO*-3	5.0 ± 0.0 ns	5.3 ± 0.5*	5.0 ± 0.0 ns	2.3 ± 0.6*	2.4 ± 0.6**
Sp RNAi *ENO*-4	5.0 ± 0.0 ns	5.1 ± 0.2 ns	5.0 ± 0.0 ns	2.3 ± 0.4*	2.3 ± 0.6*

Four T_1 progenies from independent T_0 diploid lines in *S. pimpinellifolium* (accession LA1589) background were evaluated. In each T_1 population, 10 plants containing the *NPTII* transgene were phenotyped for 10 flowers and 10 fruits each. Values are expressed as the mean ± standard deviation. Significant differences from wild-type were calculated by two-tailed Student's t-test and are represented by asterisks: $^{***}P < 0.0001$; $^{**}P < 0.001$; $^{*}P < 0.01$. ns, no statistically significant differences.

Table S5. Sequencing and mapping statistics.

Genotype	Replicate	Total reads	Mapped reads	Mapping rate (%)
RNAseq samples				
Wild-type	1	35,904,026	33,156,668	92.35
Wild-type	2	35,945,386	33,535,949	93.30
Wild-type	3	41,062,214	38,082,236	92.74
eno	1	52,784,942	49,166,224	93.14
eno	2	41,346,246	38,482,455	93.07
eno	3	34,533,698	32,225,592	93.32
DNAseq sample				
Poll of F2 wt plants		343,802,766	243,499,504	70.83
Poll of F2 *eno* mutants		354,159,746	232,338,058	65.60

Table S6. Primer sequences used for genotyping and different analysis.

Gene	Gene locus identity [a]	Primer name	Primer Sequence 5' to 3'	Purpose
SlCLV3	Solyc11g071380	EP1069	CCAATGATAATTAAGATATTGTGACG	Genotyping
		EP1070	ATGGTGGGGTTTTCTGTTCA	
		EP1071	CAGAAATCAGAGTCCAATTCCA	
LC	Solyc02g083950	lcn-SNP695-F	GTCTCTTGGATGATGACTATTGCACTTT	Genotyping
		lcn-SNP695-R	TCAGCGCCTCATTTTCTATAGTATTTGT	
		lcn-SNP695-F-cer	CTTTTCCTAAAAGATTTGGCATGAGGT	
		lcn-SNP695-R-lev	AAAGTAGTACGAATTGTCCAATCAGTCAG	
ENO	Solyc03g117230	SNP03_60481521-F	CAACAACAGCAGCGATTCCT	Genotyping
		SNP03_60481521-R	TCAAAATTCAACTCAAACGACCA	
ENO	Solyc03g117230	ENOCh03-F1q	TATTCCCTCGACCCATTTTG	qRT-PCR
		ENOCh03-R1q	AACAACAGCAGCGATTCCTT	
ENO	Solyc03g117230	ENOCh03-RNAiF	tctagactcgagCTCGACCCATTTTGGGATAA	RNAi construct
		ENOCh03-RNAiR	atcgatggtaccACACGTGCGTTTCAGAATTG	
ENO	Solyc03g117230	ENO_ish-F	TACCCTCTGCACCTTCCATC	*In situ*
		ENO_ish-R	ACACGTGCGTTTCAGAATTG	hybridization
SlCLV3	Solyc11g071380	SlCLV3_ish-F	TTTCAATCTCTTTGTCTTGCTGA	*In situ*
		SlCLV3_ish-R	CTTAGGTTTCTTAGGACTAGCACCA	hybridization
SlWUS	Solyc02g083950	SlWUS_ish-F	CGCCTCTGCCACTGATAATAA	*In situ*
		SlWUS_ish-R	GAAAAGGGTAAGTTGCTGGAGA	hybridization
ENO	Solyc03g117230	ENO Targuet-F	ATTGCTGGGTACGTTCGACACCC	CRISPR/Cas9
		ENO Targuet-R	AAACGGGTGTCGAACGTACCCAG	sgRNA
ENO	Solyc03g117230	ENO CRISPRseq-F	CTCCACAGTGACACTTACCCC	CRISPR/Cas9
		ENO CRISPRseq-R	ATCGCTGCTGTTGTTGGAGA	sequencing
ENO	Solyc03g117230	ENOy2hF	caccATGTATAATTCCCTTTCTCA	EMSA - pENTR/
		ENOy2hRs	CTACTTCCTCCTGAAGCCGA	D-TOPO vector

SlWUS	Solyc02g083950	SlWUS_EMSA-F<br>SlWUS_EMSA-R	GATCCATATAATTTTTACAAGTTGGAC<br>TGAATCATATAAATACAGTGACGGCTA	EMSA - dsDNA biotinylated probe
ENO	Solyc03g117230	ENOseq_prom-1F<br>ENOseq_prom-1R	TTGTCAAACACTTACAGAAATTAAAA<br>GCAGCGATTCCTTCAAGAAC	Haplotype sequencing
ENO	Solyc03g117230	ENOseq_prom-2F<br>ENOseq_prom-2R	ACACAAAATCCCCTTTACCC<br>TGAATAAATGGGCAGCTGAA	Haplotype sequencing
ENO	Solyc03g117230	ENOseq_prom-3F<br>ENOseq_prom-3R	TTTCCAGATTCCTCCGATTG<br>AGAAAAAGGGAAAACTCAAAACC	Haplotype sequencing
ENO	Solyc03g117230	SNP_ENO_Hap5F<br>SNP_ENO_Hap5R<br>ENO_Hap5_5’FAM<br>SNP_ENO_Hap9F<br>SNP_ENO_Hap9R<br>ENO_Hap5_5’HEX	ACAGTACAATGTATAATTCCCTT<br>GAAACAGAGTAATTATCCCAAAA<br>TGAACCAAATCGTCACCATAATTTCTCCA<br>GAAACAGAGTAATTATCCCAAAA<br>ACAGTACAATGTATAATTCCCTT<br>TGGAGAAATTACGGTGACGATTTGGT	Allele specific TaqMan ddPCR

[a] Gene locus identity based on ITAG release 2.4 official annotations on the SL2.50 genome build by the International Tomato Annotation Group (ITAG). The SOL Genomics Network (SGN) http://solgenomics.net/

SI References

1. F. Pérez-Martín, F. J. Yuste-Lisbona, B. Pineda, M. P. Angarita-Díaz, B. García-Sogo, T. Antón, S. Sánchez, E. Giménez, A. Atarés, A. Fernández-Lozano, A. Ortíz-Atienza, M. García-Alcázar, L. Castañeda, R. Fonseca, C. Capel, G. Goergen, J. Sánchez, J. L. Quispe, J. Capel, T. Angosto, V. Moreno, R. Lozano, A collection of enhancer trap insertional mutants for functional genomics in tomato. *Plant Biotechnol. J.* **15**, 1439-1452 (2017).
2. A. Fernández-Lozano, F. J. Yuste-Lisbona, F. Pérez-Martín, B. Pineda, V. Moreno, R. Lozano, T. Angosto, Mutation at the tomato *EXCESSIVE NUMBER OF FLORAL ORGANS* (*ENO*) locus impairs floral meristem development, thus promoting an increased number of floral organs and fruit size. *Plant Sci.* **232**, 41–48 (2015).
3. R. Lozano, T. Angosto, P. Gomez, C. Payan, J. Capel, P. Huijser, J. Salinas, J. M. Martinez-Zapater, Tomato flower abnormalities induced by low temperatures are associated with changes of expression of MADS-box genes. *Plant Physiol.* **117**, 91–100 (1998).
4. B. Langmead, S. L. Salzberg, Fast gapped-read alignment with Bowtie 2. *Nat. Methods* **9**, 357–359 (2012).
5. M. A. DePristo, E. Banks, R. Poplin, K. V. Garimella, J. R. Maguire, C. Hartl, A. A. Philippakis, G.del Angel G, Rivas MA, Hanna M, McKenna A, Fennell TJ, Kernytsky AM, Sivachenko AY, Cibulskis K, Gabriel SB, Altshuler D, Daly MJ. A framework for variation discovery and genotyping using next-generation DNA sequencing data. *Nat. Genet.* **43**, 491–498 (2011).
6. P. Danecek, A. Auton, G. Abecasis, C. A. Albers, E. Banks, M. A. DePristo, R. E. Handsaker, G. Lunter, G. T. Marth, S. T. Sherry, G. McVean, R. Durbin, 1000 Genomes Project Analysis Group, The variant call format and VCFtools. *Bioinformatics* **27**, 2156-2158 (2011).
7. R Development Core Team. R: A language and environment for statistical computing. R Foundation for Statistical Computing (2011).
8. K. Tamura, G. Stecher, D. Peterson, A. Filipski, S. Kumar, MEGA6: Molecular evolutionary genetics analysis version 6.0. *Mol Biol Evol.* **30**, 2725-2729 (2013).
9. M. A. Larkin, G. Blackshields, N. P. Brown, R. Chenna, P. A. McGettigan, H. McWilliam, F. Valentin, I. M. Wallace, A. Wilm, R. Lopez, J. D. Thompson, T. J. Gibson, D. G. Higgins, Clustal W and Clustal X version 2.0. *Bioinformatics* **23**, 2947-2948 (2007).
10. M. Vazquez-Vilar, J. M. Bernabé-Orts, A. Fernandez-Del-Carmen, P. Ziarsolo, J. Blanca, A. Granell, D. Orzaez, A modular toolbox for gRNA–Cas9 genome engineering in plants based on the GoldenBraid standard. *Plant Methods* **12**, 10 (2016).
11. J. C. Oliveros, M. Franch, D. Tabas-Madrid, D. San-León, L. Montoliu, P. Cubas, F. Pazos, Breaking-Cas-interactive design of guide RNAs for CRISPR-Cas experiments for ENSEMBL genomes. *Nucleic Acids Res.* **44**, W267-271 (2016).
12. S. V. Wesley, C. A. Helliwell, N. A. Smith, M. B. Wang, D. T. Rouse, Q. Liu, P. S. Gooding, S. P. Singh, D. Abbott, P. A. Stoutjesdijk, S. P. Robinson, A. P. Gleave, A. G. Green, P. M. Waterhouse, Construct design for efficient, effective and high-throughput gene silencing in plants. *Plant J.* **27**, 581-590 (2001).
13. A. P. Gleave, A versatile binary vector system with a T-DNA organisational structure conducive to efficient integration of cloned DNA into the plant genome. *Plant Mol Biol.* **20**, 1203-1207 (1992).

14. P. Ellul, B. Garcia-Sogo, B. Pineda, G. Ríos, L. A. Roig, V. Moreno, The ploidy level of transgenic plants in *Agrobacterium*-mediated transformation of tomato cotyledons (*Lycopersicon esculentum* L. Mill.) is genotype and procedure dependent. *Theor. Appl. Genet.* **106**, 231-238 (2003).

15. A. Atarés, E. Moyano, B. Morales, P. Schleicher, J. O. García-Abellán, T. Antón, B. García-Sogo, F. Perez-Martin, R. Lozano, F. B. Flores, V. Moreno, M. C. Bolarin, B. Pineda, An insertional mutagenesis programme with an enhancer trap for the identification and tagging of genes involved in abiotic stress tolerance in the tomato wild-related species *Solanum pennellii*. *Plant Cell Rep*. **30**, 1865-1879 (2011).

16. G. R. Rodríguez, S. Muños, C. Anderson, S.C. Sim, A. Michel, M. Causse, B. B. Gardener, D. Francis, E. van der Knaap, Distribution of *SUN*, *OVATE*, *LC*, and *FAS* in the tomato germplasm and the relationship to fruit shape diversity. *Plant Physiol.* **156**, 275-285 (2011).

17. J. Winer, C. K. Jung, I. Shackel, P. M. Williams, Development and validation of real-time quantitative reverse transcriptase-polymerase chain reaction for monitoring gene expression in cardiac myocytes in vitro. *Anal. Biochem*. **15**, 41-49 (1999).

18. D. Kim, G. Pertea, C. Trapnell, H. Pimentel, R. Kelley, S. L. Salzberg, TopHat2: accurate alignment of transcriptomes in the presence of insertions, deletions and gene fusions. *Genome Biol*. **14**, R36 (2013).

19. M. Lawrence, W. Huber, H. Pagès, P. Aboyoun, M. Carlson, R. Gentleman, M. T. Morgan, V. J. Carey, Software for computing and annotating genomic ranges. *PLoS Comput. Biol.* **9**, e1003118 (2013).

20. M.I. Love, W. Huber, S. Anders, Moderated estimation of fold change and dispersion for RNA-seq data with DESeq2. *Genome Biol.* **15**, 550 (2014).

21. Y. Benjamini, Y. Hochberg, Controlling the false discovery rate: a practical and powerful approach to multiple testing. *J. R. Statist. Soc. B.* **57**, 289-300 (1995).

22. Z. Du, X. Zhou, Y. Ling, Z. Zhang, Z. Su, agriGO: a GO analysis toolkit for the agricultural community. *Nucl. Acids Res.* **38**, W64-W70 (2010).

23. D. M. Goodstein, S. Shu, R. Howson, R. Neupane, R. D. Hayes, J. Fazo, T. Mitros, W. Dirks, U. Hellsten, N. Putnam, D. S. Rokhsar, S. James, Phytozome: a comparative platform for green plant genomics, *Nucleic Acids Res.* **40**, D1178-1186 (2012).

24. G. Bindea, B. Mlecnik, H. Hackl, P. Charoentong, M. Tosolini, A. Kirilovsky, W. H. Fridman, F. Pagès, Z. Trajanoski, J. Galon, ClueGO: a Cytoscape plug-in to decipher functionally grouped gene ontology and pathway annotation networks. *Bioinformatics* **25**, 1091-1093 (2009).

25. M. Kearse, R. Moir, A. Wilson, S. Stones-Havas, M. Cheung, S. Sturrock, S. Buxton, A. Cooper, S. Markowitz, C. Duran, T. Thierer, B. Ashton, P. Meintjes, A. Drummond, Geneious Basic: An integrated and extendable desktop software platform for the organization and analysis of sequence data. *Bioinformatics* **28**, 1647-1649 (2012).

26. N. Kamitaki, C. L. Usher, S. A. McCarroll, "Using Droplet Digital PCR to Analyze Allele-Specific RNA Expression" in Digital PCR, Methods in Molecular Biology, Vol. 1768, G. Karlin-Neumann, F. Bizouarn, Eds. (Springer, 2018), pp. 401-422.

27. G. Zhu, S. Wang, Z. Huang, S. Zhang, Q. Liao, C. Zhang, T. Lin, M. Qin, M. Peng, C. Yang, X. Cao, X. Han, X. Wang, E. van der Knaap, Z. Zhang, X. Cui, H. Klee, A. R. Fernie, J. Luo, S. Huang, Rewiring of the fruit metabolome in tomato breeding. *Cell* **172**, 249-261 (2018).

28. The Tomato Genome Consortium. The tomato genome sequence provides insights into fleshy fruit evolution. *Nature* **485**, 635-641 (2012).

29. H. Li, B. Handsaker, A. Wysoker, T. Fennell, J. Ruan, N. Homer, G. Marth, G. Abecasis, R. Durbin, 1000 Genome Project Data Processing Subgroup, The Sequence alignment/map (SAM) format and SAMtools. *Bioinformatics* **25,** 2078-2079 (2009).

30. J. Blanca, J. Montero-Pau, C. Sauvage, G. Bauchet, E. Illa, M. J. Díez, D. Francis, M. Causse, E. van der Knaap, J. Cañizares, Genomic variation in tomato, from wild ancestors to contemporary breeding accessions. *BMC Genomics* **16**, 257 (2015).

31. C. C. Chang, C. C. Chow, L. C. Tellier, S. Vattikuti, S. M. Purcell, J. J. Lee, Second-generation PLINK: rising to the challenge of larger and richer datasets. *Gigascience* **4**, 7 (2015).

32. E. Paradis, J. Claude, K. Strimmer, APE: Analyses of Phylogenetics and Evolution in R language. *Bioinformatics* **20**, 289-290 (2004).

33. G. Yu, D. K. Smith, H. Zhu, Y. Guan, T. T. Y. Lam, ggtree: an R package for visualization and annotation of phylogenetic trees with their covariates and other associated data. *Methods Ecol. Evol.* **8**, 28-36 (2017).

34. C. Xu, K. L. Liberatore, C. A. MacAlister, Z. Huang, Y. H. Chu, K. Jiang, C. Brooks, M. Ogawa-Ohnishi, G. Xiong, M. Pauly, J. Van Eck, Y. Matsubayashi, E. van der Knaap, Z. B. Lippman, A cascade of arabinosyltransferases controls shoot meristem size in tomato. *Nat. Genet.* **47**, 784-792 (2015).

35. J. T. Robinson, H. Thorvaldsdóttir, W. Winckler, M. Guttman, E. S. Lander, G. Getz, J. P. Mesirov, Integrative genomics viewer. *Nat. Biotechnol.* **29**, 24–26 (2011).

36. U. Bodenhofer, E. Bonatesta, C. Horejš-Kainrath, S. Hochreiter, msa: an R package for multiple sequence alignment. *Bioinformatics* **31,** 3997-3999 (2015).
